# A System Computational Model of Implicit Emotional Learning

**DOI:** 10.3389/fncom.2016.00054

**Published:** 2016-06-14

**Authors:** Luca Puviani, Sidita Rama

**Affiliations:** ^1^Department of Engineering “Enzo Ferrari”, University of Modena and Reggio EmiliaModena, Italy; ^2^Local Health Unit of ModenaModena, Italy

**Keywords:** amygdala, classical conditioning, emotional learning, evaluative conditioning, misattribution, prediction error, PTSD, UCS revaluation

## Abstract

Nowadays, the experimental study of emotional learning is commonly based on classical conditioning paradigms and models, which have been thoroughly investigated in the last century. Unluckily, models based on classical conditioning are unable to explain or predict important psychophysiological phenomena, such as the failure of the extinction of emotional responses in certain circumstances (for instance, those observed in evaluative conditioning, in post-traumatic stress disorders and in panic attacks). In this manuscript, starting from the experimental results available from the literature, a computational model of implicit emotional learning based both on *prediction errors computation* and on *statistical inference* is developed. The model quantitatively predicts (a) the occurrence of evaluative conditioning, (b) the dynamics and the resistance-to-extinction of the traumatic emotional responses, (c) the mathematical relation between classical conditioning and unconditioned stimulus revaluation. Moreover, we discuss how the derived computational model can lead to the development of new animal models for resistant-to-extinction emotional reactions and novel methodologies of emotions modulation.

## 1. Introduction

In this manuscript, starting from a review and the analysis of the main experimental results in the field of implicit emotions, a novel interpretation of *associative learning* and *UCS revaluation* (and of their unavoidable interactions) is derived first. UCS revaluation represents the updating of the expected outcome (or biological value) associated with a given source of stimulation. In particular, if an UCS elicitation determines a greater (smaller) central nervous system (CNS) response with respect to the expected outcome, the value associated with the considered UCS will be increased (decreased) determining an inflation (deflation) process (Rescorla, 1974; Davey, 1989; Hosoba et al., 2001; Gottfried and Dolan, 2004; Schultz et al., 2013). Classical Conditioning occurs when an initial neutral stimulus (in other words a stimuli unable to activate the innate emotional system, so that it does not elicit emotional reactions, for instance a neutral sound) becomes paired to another stimuli, UCS, which elicits a biological relevant response, termed unconditioned response, UCR. After few CS-UCS pairings, the initial neutral CS becomes able to elicit a biologically relevant response, denoted conditioned response (CR) “similar” and generally speaking smaller than UCR (Fanselow and Poulos, 2005). In the literature CC and UCS revaluation are considered two independent learning mechanisms (Rescorla, 1974; Hosoba et al., 2001; Gottfried and Dolan, 2004). In this manuscript, considering that almost all the experiments reported in the technical literature about implicit emotional learning involve discrete trials stimulation (e.g., electric shock delivery, food delivery) and measures (neuronal activity recordings, fMRI measures, or behavioral), the derived theory and model are initially defined in a discrete time scale. The proposed model is able to justify experimental results not predictable by other existing models, and it can be adopted for the study of important paradigms, such as the Iowa Gambling Task (Bechara et al., 1994; see Section 3.3). Furthermore, starting from the obtained discrete time model its continuous time counterpart is derived next. The derivation of such a continuous time model is based on mathematical considerations and engineering standard methods under the constraints imposed by the functional connectivity between the different brain regions involved in automatic emotional processing. A dynamical continuous time model which accounts for both (a) statistical/associative learning and pattern recognition and (b) for a time-varying stimulation intensity (i.e., implicit UCS revaluation) and the consistent related phenomena (e.g., the so called *emotional contrast effect*) has not been developed yet from our knowledge. This could be due to different reasons: first of all UCS revaluation has not obtained much attention over years and the researches have been focused mainly in CC; second, CC is intrinsically time-discrete. Nevertheless, the above cited continuous model is useful because shows the dynamics which lead to the updating of the emotional value over time, due for instance to a time-varying stimulation which exerts alternations of both aversive and appetitive values (for instance, a stimulation can elicit a slow aversive increase of tension and then a fast tension release, inducing a given organism to perceive it as an appetitive source of stimulation since it produces emotional rewarding effects). Indeed, classical conditioning model cannot describe the frequency or time emotional response under the influence of a time-varying stimulation, such an acoustic signal which varies between appetitive and aversive response induction (for instance varying both the sound frequency and intensity), as occurs in music. More specifically, a continuous time dynamical model can show how the emotional system *tracks* a given source of stimulation, either if such a source elicits the organism through an information flux (in other words the emotions are induced by aversive and appetitive information such as smiles or angry facial expressions, or a movie, but not by exerting a physical or energy based interactions) or through an energy based flux (i.e., through a stimulation due to energy exchange between the stimulus and the organism's receptors, such as a painful stimulation).

**Figure 1:**
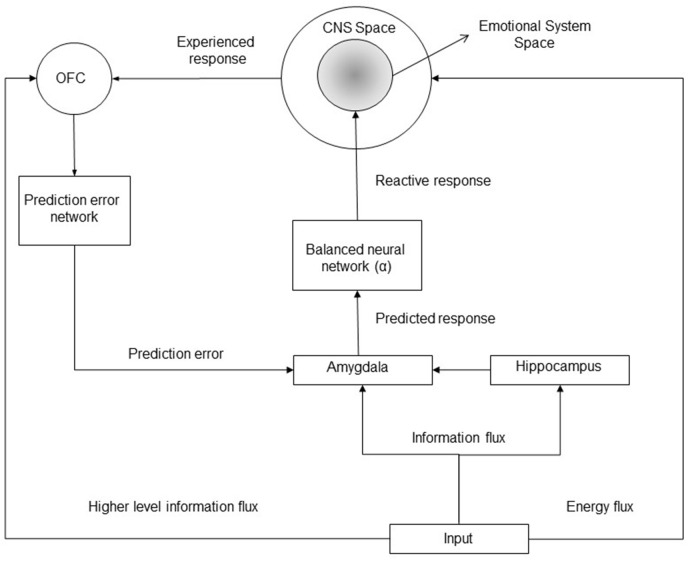
**Block diagram of the implicit emotional system**. A given input to the emotional system can provide (1) an energy flux determining a cascade of reaction which finally elicit a CNS response (an increase/decrease of the mean firing rates of neuronal populations within the central nervous system); (2) an information flux determining a stimulus identification within the amygdala and within the orbitofrontal cortex (OFC); furthermore, specific higher cognitive level information can be processed by the OFC only. OFC encodes the biological predicted or expected outcome for a given stimulus, it manages the computation of prediction errors updating the expected emotional response (and the reactive response) within the amygdala. The amygdala elicits emotional neuronal populations with an intensity proportional to the expected response.

It is worth noting that emotional learning models which do not account for the implicit intensity stimulation evaluation (i.e., the UCS evaluation and revaluation over time or over trials) cannot predict or justify important psychophysiological phenomena which originates from specific dynamics of the emotional arousal. Such phenomena are the so called resistant-to-extinction (or inextinguishable) emotional responses, such as those observed in *evaluative conditioning* or in pathological reactions observed in panic attacks (Meuret et al., 2006) and post traumatic stress disorder (PTSD) (Beck and Sloan, 2012; Parsons and Ressler, 2013; Perusini et al., 2016). More specifically, emotional learning models based on associative learning (i.e., CC; Pavlov, 1927) account for the conditioned stimulus (CS) response variation due to the modulation of the statistical contingencies between an actively eliciting stimulus (called unconditioned stimulus, UCS) and the CS itself (which was neutral before the CS-UCS pairing), but they cannot say nothing about the intensity dynamics associated with the given UCS (which represents the causal source of stimulation). In other words, CC-based models describe the CS-UCS connection strength neglecting the relation between the UCS representation and the expected response associated with it (i.e., unconditioned response, UCR), which, in turn, may depend also (and indirectly) on the CS-UCS connection strength (see Figure 1). For these reasons, these models, cannot say nothing about (1) the neuronal populations involved in CS response (CR); (2) the mathematical expression of the intensity of the CR at the end of the acquisition process (in other words the CR intensity when CS predicts with absolute certainty the occurrence of UCS); it worth mentioning that until now the qualitative explanation is that the “*CR is similar but smaller than the UCR*” (Fanselow and Poulos, 2005); (3) the mathematical expression of UCR. The theory developed in this manuscript shed lights on these points, and, doing this, it will be able to justify how resistant-to-extinction emotional responses originate. Furthermore, the model permits the development of stimulation functions able to induce PTSD-like emotional reactions in animal models, or for emotional modulation (for instance decreasing an emotional response).

The manuscript is organized as follows. In the Paragraph “Materials and Methods” some fundamental definitions and concepts are provided and motivated first; thereafter, a review of the technical literature about empirical results and models on implicit emotional learning is presented, together with qualitative and quantitative considerations, which permit the development of the structure of the models (more specifically the description of the main brain regions involved and their functional role and connections) and, successively, the quantitative relations and constraints between the involved variables (more specifically the linearity hypothesis, the relation between a predicted/expected outcome and the reactive response, the *integration property* and the *emotional contrast effects*); successively, the main assumptions and hypothesis of the model are provided and motivated; in the subsequent Sections the model is developed in three different cases: (a) UCS revaluation in the discrete time scale; (b) joint CC and UCS revaluation in the discrete time scale; (c) continuous time scale. In the “Results” Section the main post-predicted results of the models and quantitative justification of specific psychophisiological phenomena are presented together with the comparison with existing models; furthermore, a Section on the validation, interpretation and applicability of the derived models and theory closes the Paragraph. Finally, the “Discussion” Section closes the manuscript.

## 2. Materials and methods

### 2.1. Definitions and concepts

#### 2.1.1. Motivation

This Section provides fundamental concepts and definitions for the development of the model (some of the following definitions and analysis are taken from Puviani et al., 2016). First a mathematical definition of CNS response is given, such a definition is useful for the subsequent definition of emotional (and reactive) response. These definitions are needed since, in CC theory and models, emotional responses (or reactions) are not well defined from a quantitative perspective; indeed in CC some behavioral or autonomic correlated responses are measured during experiments, such as the degree of salivation or the indirect measure of the arousal (i.e., the overall intensity of an emotional response) through the skin conductance response (SCR) evaluation. It is worth noting that such indirect measures always reflect a CNS response. Furthermore, the definition of *source of stimulation* is provided, since in CC theory the distinction between CS and UCS is based on the fact that an UCS exerts an innate reaction and the CS does not; nevertheless, this differentiation is not always satisfactory, since a previously neutral stimulus could became an UCS in certain circumstances. Moreover, the differentiation of two different types of stimulation are specified, these are the *active* and *reactive* stimulations; conversely in CC theory and derived models this distinction is not considered, so that it is not possible to express the overall CNS response as the contribution of the two quantities which, generally speaking, can vary independently during emotional learning (indeed, the brain integrates the two contributions, so that they becomes indistinguishable from a neurophysiological perspective, but the analytical distinction is useful from a model perspective). Finally, the definition of *reactive mimicking* property is provided and it is based on empirical evidences from pharmacological conditioning experiments. Such a property shed lights on the emotional (and, generally speaking, the reactive) learning mechanism, evidencing that whenever an UCS stimulates a CNS response, the brain stores the reactive response (i.e., the intensity and the elicited CNS sub-population) which will be associated with the given UCS for future predictions.

#### 2.1.2. Definitions

##### 2.1.2.1. CNS response

A generic response induced within the CNS can be represented by the superposition of the activity of different neural populations; more specifically, assuming that the CNS consists of *N* different populations, the response, denoted with the vector **y**, can be expressed as:

(1) $$\begin{array}{l} \text{y}=\overset{N}{\underset{i=1}{\sum }}y_{i}{\text{v}}_{i}, \end{array}$$

where *y*_*i*_ represents the *i*-th neuronal population activity and {**v**_*i*_; *i* = 1, 2, …, *N*} represents a set of versors, being associated with different neuronal populations, which form a complete basis B for the CNS space. More specifically, *y*_*i*_ is a real quantity representing the product between the mean number of elicited neurons and their mean firing rates for the *i*-th neuronal population (with *i* = 1, 2, …, *N*); consequently, *y*_*i*_ takes on a positive (negative) value if the response produces an increase of (a decrease or inhibition of) the activity for the *i*-th population, and is equal to zero whenever the response does not involve any adjustment for the baseline activity of the population. It is worth noting that the different neuronal populations could be interdependent (i.e., B does not represent an orthonormal basis).

##### 2.1.2.2. Source of stimulation

A “source of stimulation” is defined as any stimulus able to causally and directly induce a CNS response (e.g., a painful stimulation). Some sources of stimulation (more specifically their neural representations) are natively coded within the mammalian brain, shaped by evolution (Ohman, 1993; Ohman and Soares, 1993; Esteves et al., 1994), while others are acquired through experience (Flykt et al., 2007); nevertheless a conditioned stimulus does not represent a source of stimulation, since it cannot causally and directly determine a CNS response, instead it may signal an imminent stimulation of a given UCS, and, for this reason, it can indirectly determine a CNS response. It can be inferred from experimental results based on subliminal stimulation (Ohman and Soares, 1993) that encoding a stimulus as a source of stimulation (i.e., as the responsible of the elicitation) or as contextual or conditioned stimulus, makes the difference in the determination of the specific brain region in which it will be stored; more specifically only the sources of stimulation are stored in the *basolateral amygdala* (BLA) in a rapid-access region, elicitable through the thalamo-amygdala pathway, while CSs do not. The terms “source of stimulation” and “UCS” are adopted indiscriminately in the following.

##### 2.1.2.3. Active stimulation and active response

An active stimulation is defined as any stimulation causally and directly exerted by an UCS *through an energy flux* (e.g., mechanical, thermal, chemical, pharmacological…) exchanged between the UCS itself and a given organism. Whenever an UCS exerts an active stimulation the resulting elicited CNS response is causally and directly related to the intensity of the energy flux (and its temporal derivatives) transferred from the source of stimulation to the organism's receptors. For instance a painful thermal stimulus exerts an active painful elicitation transferring heat to specific receptors of the given organism; furthermore if the transferred heat increases the perceived painful response will increase too.

##### 2.1.2.4. Reactive stimulation and reactive response

A reactive stimulation is defined as any stimulation induced within the CNS exclusively *through an information flux*. Thus, a reactive stimulation exerts its action through information processing and not by a direct energy flux. For instance, a CS previously paired with a given UCS induces a CNS response (e.g., fear) through its mere perception (i.e., information processing) and not because energy flux transfer toward the organism. It is important pointing out that, obviously, the mere perception of a stimulus (e.g., a CS) is sustained by a certain energy flux, such as acoustic (mechanical) or light intensity variation (electromagnetic), nonetheless, in this case, the response induced within the CNS is not causally and directly determined by the energy flux, or, in other words, the response is not directly related to the intensity of the energy flux, instead here the energy represents a mean to transfer an information flux. Indeed, a CS, may determine a CNS response because the information flux revealing its presence triggers a previously learned response. Generally speaking, a reactive response consists of a “self-induced” reaction triggered by an information flux (e.g., by a visual, auditory, olfactory, gustatory perception or by imagination), conversely, an active response is sustained by an external energy flux (e.g., an active drug or an electric shock). It is possible to exert both active and reactive stimulations concurrently; for instance, an hidden drug administration exerts only an active stimulation, since a pharmacological (chemical) flux is provided while no information is given, conversely, an open drug administration may induce both an active pharmacological response and a reactive stimulation due to cognitive (and even unconscious or imaginary) information processing (Amanzio and Benedetti, 1999; Benedetti et al., 2003; Benedetti, 2008).

On the basis of the above mentioned definitions it follows that an UCS can exert both an active and a reactive stimulation, while a CS can induce only a reactive stimulation.

##### 2.1.2.5. Reactive (emotional) system

Generally speaking, a reactive stimulation cannot involve all the CNS neural components, since, for instance, a somatosensory stimulation can occur only through an energy flux (e.g., mechanical) and not by a simple information processing; for this reason only a “sub-space” of the CNS neuronal populations can be reactively elicited. The CNS sub-space which can be elicited through a reactive (information flux based) stimulation is termed reactive system (as will be clarified in the following the emotional system represents a sub-space of the reactive system). Hence, provided that *N* denotes the number of the distinct neuronal populations within the CNS (Equation 1), and that *K* denotes the number of the reactive system neural populations, it follows that *K* ∈ *N*. Which are the neuronal components within the CNS belonging to the reactive system? The answer comes from classical conditioning and pharmacological conditioning experiments in which a CS exerts a reactive stimulation after being paired with an active UCS. From the technical literature emerges that the reactive system may involve: (1) emotional responses (which include, for instance, the dopaminergic mesolimbic and mesocortical system, Scott et al., 2007; Colloca, 2014; the fear and anxiety related circuits, McNally et al., 2011; Li and McNally, 2014; the endocannabinoid and opioid system in placebo analgesia, De Pascalis et al., 2002; Petrovic et al., 2002; Zubieta et al., 2005; Wager et al., 2007; Eippert et al., 2009; Watson et al., 2009; Nolan et al., 2012, the serotoninergic system, the target neuronal systems of depression, anxiety and addiction; see Benedetti, 2008); (2) the dopaminergic motor system (De la Fuente-Fernandez et al., 2001; De la Fuente-Fernandez and Stoessl, 2002); (3) the *humoral immune response system* (in particular the components of the CNS such as the hypothalamic-pituitary-adrenal axis, HPA, or the sympathetic nervous system, SNS; Goebel et al., 2002; Cacioppo et al., 2007; Benedetti, 2008; Vits et al., 2011); (4) the endocrine system; (see Benedetti, 2008; Enck et al., 2008 for a review).

##### 2.1.2.6. Reactive (and emotional) mimicking

From a growing body of literature (Amanzio and Benedetti, 1999; Petrovic et al., 2002; Haour, 2005; Eippert et al., 2009; Guo et al., 2010; Lui et al., 2010; Nolan et al., 2012) it is reported that pharmacological conditioning determines a reactive response which mimic the active pharmacological response. The above mentioned property is termed here reactive mimicking. For instance, experimental results reported in Ito et al. (2000) show that an increase in dopamine release in the *ventral striatum*, measured through microdialysis, are observed not only when rats self administer cocaine (UCS), but also when they are solely presented with a tone (CS) that has been previously paired with cocaine administration. Furthermore, provided that the reactive system represents only a subset of the CNS, it is evident that only such a subset of the CNS neuronal populations can be mimicked. For instance, a CS previously paired with a painful UCS stimulation will be able to elicit only a specific portion of the components that were actively stimulated by the UCS; such components represent the emotional response (e.g., the activation of anterior cingulate cortex and the anterior insula; Singer, 2004), and, they cannot involve the somatosensory neural populations, even if these were involved in the original UCR.

### 2.2. Derivation of the emotional dynamical system structure

In this Section the role of the key brain regions involved in emotional processing and response are reviewed from the literature. The purpose of this Section is to infer the functional structure of the dynamical emotional system.

#### 2.2.1. Emotional responses, amygdala and orbitofrontal cortex

In mammalian brains the amygdala represents the core center in the formation and storage of emotional events and in the elicitation of emotional responses. In particular, in a growing body of literature (Schoenbaum et al., 1999; Glascher and Adolphs, 2003; Paton et al., 2006; Choi and Jeansok, 2010; Amano et al., 2011; Sangha et al., 2013) it is shown that amygdala is necessary for fear responses, and that no reactive fear responses are instantiated in the absence of an intact amygdala (Choi and Jeansok, 2010). Furthermore, the amygdala mediates both appetitive (i.e., rewarding) and aversive stimuli (Muramoto et al., 1993; Schoenbaum et al., 1999; Paton et al., 2006; Shabel and Janak, 2009; Amano et al., 2011; Sangha et al., 2013; Gore et al., 2015); in the former case the *basolateral amygdala* (BLA) neurons project onto the nucleus accumbens (NAcc), whereas in the latter one onto the *centromedial amygdala* (CeM) (Namburi et al., 2015). Hence the amygdala represents the key region for the elicitation of any reactive emotional response and it elicits (both directly or indirectly) emotional and motivational areas of the brain (LeDoux, 2000; Sah et al., 2003; Gore et al., 2015; Janak and Tye, 2015; Tovote et al., 2015). Nevertheless, it is worth noting that if the amygdala is damaged, an active elicited response (e.g., an unconditioned painful stimulus) can be still elicited. Moreover, experiments performed adopting optogenetic manipulations have evidenced that the representation of any UCS is stored within the BLA (Redondo et al., 2014; Gore et al., 2015). However, further fMRI studies (Gottfried et al., 2003; Gottfried and Dolan, 2004; O'Doherty, 2004; Kringelbach, 2005; Dolan, 2007; Pessoa, 2010) have shown that UCS representations (and its associated “biological values” or, in other words, the outcome which is expected from the given UCS) are encoded not only within the amygdala, but also in the orbitofrontal cortex (OFC). The fact that a stimulus representation and its associated expected outcome are stored in different brain regions (i.e., duplicated) could seem a waste of resources; nevertheless different reasons could justify this redundancy. Indeed, on the one hand it is important that the representation of relevant stimuli (such as fear relevant stimuli) are accessible through rapid access pathways, such as thalamo-amygdala pathway (LeDoux, 1996, 2000; Ohman, 2005), promoting a quick reaction whenever the stimulus is perceived. On the other hand, it is also important that a stimuli representation can be integrated with relevant cognitive information (when available) for the inference or prediction of the probable outcome. For instance, animals may learn that a given stimulus (e.g., a predator) is threatening observing others facing with it (Olsson et al., 2007; Olsson and Phelps, 2007), without the need of experiencing directly a stimulus elicitation. Hence, the OFC integrates different pieces of information (especially higher level cognitive ones) for inferring a probable outcome, and to update the response associated with the given UCS in “faster” subcortical regions (i.e., in the amygdala). Furthermore, prefrontal regions, like the dorsolateral prefrontal cortex (DLPFC), may interact with OFC to enhance or inhibit the response elicited by the amygdala (Ohman, 2005; Dolan, 2007). For instance, initial amygdala response to a fear-relevant but non-feared stimulus (e.g., pictures of spiders for a snake phobic) disappears with conscious processing by the activation of DLPFC and OFC (Ohman, 2005). Furthermore, also experiments in the filed of decision making have evidenced that OFC supervises the amygdala (Wallis, 2007; Rolls and Grabenhorst, 2008; Kennerley and Walton, 2011). Finally, it is worth pointing out that OFC is not necessary for classical conditioning, however, it is certainly needed for modifying the response if the predicted outcome is revaluated (i.e., UCS inflation and devaluation; Gallagher et al., 1999; Stalnaker et al., 2015).

#### 2.2.2. Error-driven learning

From a growing body of literature emerges that learning occurs through the computation of specific *error-signals* (or *prediction errors*) (Schultz and Dickinson, 2000; Garrison et al., 2013). Generally speaking, the prediction error is defined as the difference between the response (or the outcome) expected from a given stimulation and the response actually perceived by the elicited organism. This definition relies on experimental observations acquired in functional imaging studies (Berns et al., 2001; O'Doherty et al., 2003; Garrison et al., 2013), or directly measured in dopaminergic circuits (e.g., in the *ventral tegmental area*, VTA) or in other fear-related circuits (Schultz, 2000, 2006; Schultz and Dickinson, 2000; Waelti et al., 2001; Bray and O'Doherty, 2007; Delgado et al., 2008; McNally et al., 2011; Steinberg et al., 2013; Li and McNally, 2014).

Different mathematical models describing classical conditioning learning (e.g., Rescorla-Wagner model, Rescorla and Wagener, 1972; Miller et al., 1995, or *temporal difference* (TD) *models*, Sutton, 1988; Sutton and Barto, 1990; Schultz et al., 1997; O'Doherty et al., 2003), or describing learning in general, such as the probabilistic (Bayesian) “perception” and “action” learning models (i.e., the *predictive coding* (PC) (Friston, 2003, 2008) and *active inference model* (Friston et al., 2009, 2010), assume that coding behavioral responses involves the computation of a prediction error. More specifically, the brain makes predictions in relation to a given stimulus and, on the basis of the experienced outcome, the prediction is updated through the prediction error. If the experienced outcome is greater (lower) than the prediction, the computed error signal is positive (negative) and corrects the new prediction; furthermore, if the experienced response coincides with the expected outcome, the error signal is zero and no prediction updatings take place.

#### 2.2.3. On the computation of the prediction errors

A growing body of literature (Schultz, 1998, 2000; Waelti et al., 2001; Schultz, 2006; Delgado et al., 2008; Bourdy and Barrot, 2012) evidenced that in emotional learning, populations of dopaminergic neurons encode prediction errors evaluating the difference between what is expected (i.e., the expected reward) and what is really occurring; furthermore, the prediction error is exploited to correct and modulate the individual's emotional and behavioral response. The prediction error computed in these dopaminergic regions can be positive or negative and can drive appetitive or aversive emotional reactions (Delgado et al., 2008).

It is not completely clear if prediction errors driving emotional responses are evaluated in different brain regions, depending on the nature of the involved emotional neuronal populations, or if dopamine neurons encode prediction errors related to all the involved populations; however, in the computation of the emotional error signal, a fundamental role is played by the OFC (O'Doherty, 2007). In fact, various experimental results have evidenced that the OFC generates information about expected outcomes which are deemed critical in the computation of prediction errors (e.g., see Takahashi et al., 2009 and references therein) and these results are consistent with the relation between the reward-related activity in OFC and VTA dopamine neurons (Takahashi et al., 2009). Experimental results have also evidenced that, when OFC and midbrain data are juxtaposed, anticipatory activity observed in the OFC is inversely related to dopaminergic error signaling downstream (Stalnaker et al., 2015). This suggests that the error signals in other brain areas might depend partly on OFC input for properly calculating the errors (Schoenbaum et al., 2009; Stalnaker et al., 2015).

#### 2.2.4. The role of the hippocampus in emotional learning and biological and functional differences between UCS revaluation and classical conditioning

As reviewed above, the amygdala encodes the representation of UCSs and the related emotional responses; furthermore, it is well known that contextual information and statistical contingencies associated with a given UCS are encoded by the hippocampus (Bechara et al., 1995; Richardson et al., 2004). Important questions arise: what is the functional connectivity between a CS stored in the hippocampus and an emotional response? Does the hippocampus store emotional responses associated with the CSs? Which is the functional connection between the hippocampus and the amygdala? Responding to these questions permits to elucidate the role of the hippocampus in emotional learning and to differentiate the two learning mechanisms: CC and UCS revaluation. Such responses come from recent optogenetic experimental results (Redondo et al., 2014; Gore et al., 2015) which have evidenced that the hippocampal engram memory (which codes a CS) is neutral and could freely associate with either positive or negative emotions, through the UCS representation coded within the BLA. Furthermore, optogenetic reactivation of the hippocampal *dentate gyrus* (DG) engram cells coding a CS, during the presentation of a new UCS having valence opposite to the original UCS (which was previously paired with the CS itself), strengthens the connectivity of these cells with the new subset of the BLA neurons, while weakening the connections established during the original learning process. In other words, the simultaneous activation of a CS neural representation and of a new UCS strengthens a CS-UCS synaptic connection and, at the same time, weakens the connection between the CS and the previously associated UCS, which is not simultaneously active. These results evidence three important features: (1) a CS stored in the hippocampus has to be connected to an UCS representation within the BLA in order to trigger an emotional response; (2) the CS-UCS connection can be strengthened or weakened through synaptic Hebbian plasticity (i.e., through the mechanism “cells that fire together wire together,” without the need of error signal computations or UCS revaluation); (3) the fact that the CS engram memory is emotionally neutral it means that the emotional reaction triggered whenever it is perceived is exclusively due to the CS-UCS *synaptic strength*, denoted ω_*CS*−*UCS*_ in the following. In turn, the UCS representation is associated with an emotional value (denoted *i*_*R*_ in the following). Hence, the term *i*_*R*_ represents the *reactive response* triggered whenever a CS connected with the given UCS is perceived.

Other experimental evidences support the fact that CC is not driven by prediction errors (see Section 3.2.4).

#### 2.2.5. Functional connectivity of the implicit emotional system

On the basis of the reviewed results in the previous Sections (more specifically see Sections 2.2.1–2.2.4) the functional connectivity of the brain regions involved in the implicit emotional learning can be inferred (see Figure 1). In particular, it is shown that a given stimulation can elicitate both an information and an energy flux, more specifically, the energy flux determines a direct response within the CNS system (for instance a painful stimulation determines an increasing firing rates of the neurons belonging to the insula, the anterior cingulate cortex, the sensorimotor cortex, and others), while the information flux can be processed by the amygdala (e.g., by a mere stimulus perception), by the hippocampus (for statistical and contextual recognition) and by the OFC (which can process higher level and structured information). The amygdala elicits a reactive response onto the emotional system (which involve only a sub-population of the entire CNS neuronal populations), and such a response can be modulated and corrected through the error signals whose computation is managed by the OFC.

**Figure 2:**
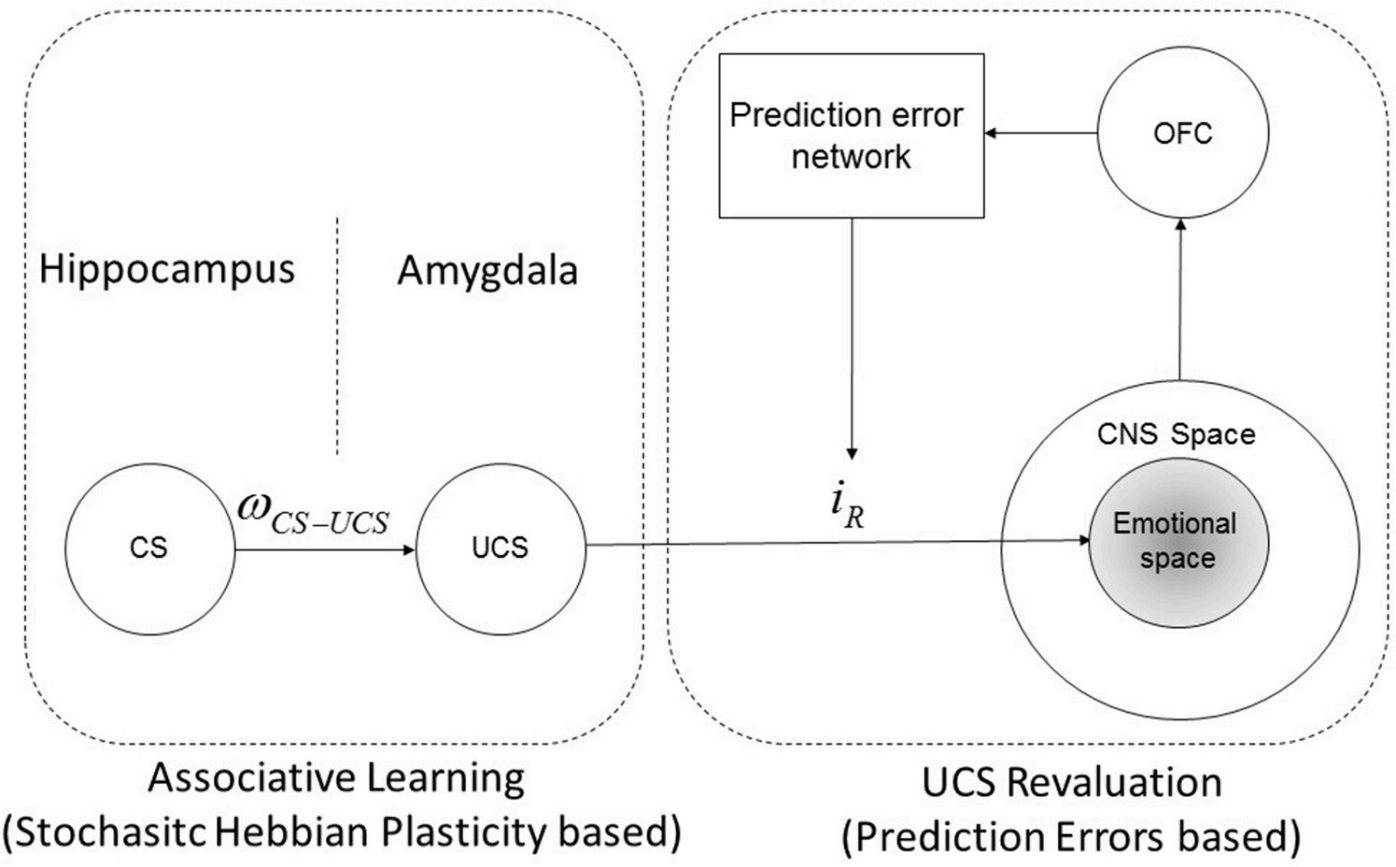
**CS-UCS-emotional response diagram**. Schematic representation of the synaptic connections between a CS, the associated UCS and the reactive response (*i*_*R*_) associated with the representation of the UCS itself. The reactive response is determined and modulated by UCS revaluation learning, instead the connection strength between CS and UCS (ω_*CS*−*UCS*_) is determined by classical conditioning learning. The reactive response acts within a central nervous system sub-space called emotional space, which involve the emotional neuronal populations. Note that this schematic representation is in agreement with the experimental results shown in Gore et al. (2015) and Redondo et al. (2014).

Moreover, the reviewed results in Section 2.2.4 permits to infer the representation of CC and UCS revaluation as sketched in Figure 2. In particular, it is shown that UCS is stored within the amygdala, which in turn projects (through direct and indirect systems as reviewed in Section 2.2.1) onto the emotional system within the CNS; furthermore, the amygdala response is modulated by prediction errors with a feedback loop, which determines the UCS revaluation. On the other hand the CS (which, depending on the type, can be stored within the hippocampus or even in a region of the amygdala different from the region which contains the UCS representations) is not directly associated to an emotional response, but it is connected with an UCS representation, whose connection strength is denoted ω_*CS*−*UCS*_. Moreover, it can be shown that UCS revaluation does not change the CS-UCS synaptic connection strength, conversely, as it is clarified in (Section 2.5.2; see also “Supplementary Material”) an increase of CS-UCS connection strength leads inevitably to an UCS inflation until *i*_*R*_ reaches an asymptotic value.

#### 2.2.6. Attribution of a source of emotional stimulation and predictive coding

In the previous Sections it has been shown that a given UCS representation within the BLA is being associated with a specific reactive response which can also be modulated through prediction errors (see Figure 2). In order to build such a structure, the brain has to infer which is the stimulus that generates the CNS response. For instance, a device eliciting a painful response will be encoded as an UCS since it can be easily detected and attributed as the causal *source* of the painful stimulation; in this case the source of stimulation has been attributed correctly. However, whenever an emotional response due to a source of stimulation is attributed to a “wrong” source, an event of *source misattribution* occurs (Cotton, 1981; Bryant, 2003; Jones et al., 2009 and references therein). It is worth pointing out that misattribution may result either from conscious, accessible and measurable controlled processes, or from spontaneous, inaccessible, automatic processes (Uleman, 1987; Anderson, 1989). In the last case this phenomenon is called *implicit misattribution* (Uleman, 1987; Anderson, 1989; Hutter and Sweldens, 2013). Generally speaking, the brain is an inference machine that actively predicts and explains its sensations; more specifically, the brain tries to explain the cause of its sensations through a probabilistic model (Friston, 2010). This concept is at the basis of the Bayesian brain hypothesis and of the so called *predictive coding* theory (Friston, 2008), which shows how automatic inference about the causes of sensory inputs are performed in a hierarchical structure within the brain. What is important in the development of our model is that *UCS attribution*is based on complex hierarchical and recursive (feedforward and backward) signals propagation between different layers, which generate a *probabilistic* model and representation about the cause(s) of the stimulation. This means that in the structure of the model we derived (see Figure 2), the UCS-*i*_*R*_ association is subject to eventual misattributions, and that two or more UCS representations could be associated to a (shared) given response if the brain fails to correctly infer the actual eliciting UCS; furthermore, in a limit case, a neutral and irrelevant stimulus could be attributed as the source of stimulation (i.e., a misattribution occurs). The attribution and misattribution phenomena can be quantitatively considered in the general structure of our model. In fact, it can be assumed that the reactive response the brain associates to a given UCS is proportional to the degree of *cause attribution belief* the brain predicts for that UCS. For instance, in the presence of two possible eliciting stimuli, the brain can infer all the possible probability attributions between the ranges (0–100%) and (100–0%), and, such a cause probability assignment is determined by (a) Bayesian prior belief distributions and (b) the actual stimulation conditions and perceptions. The attribution and misattribution phenomena will play an important role for the quantitative explanation of *evaluative conditioning* (see Section 3.2.3).

### 2.3. Quantitative relations and constraints between variables

In this Section the main quantitative/mathematical assumptions and the relations between the variables involved in implicit emotional learning are inferred by a review of the literature and through analytical considerations.

#### 2.3.1. Linearity hypothesis

Recent computational and *in vivo* analysis have evidenced that cortical circuit have recurrent excitatory and inhibitory connections (van Vreeswijk and Sompolinsky, 1996, 1998; Doiron and Litwin-Kumar, 2014; Pehlevan and Sompolinsky, 2014; Deneve and Machens, 2016). Such a network architecture comprises excitatory and inhibitory neuronal populations, and the connectivity could be random and sparse. Computational studies about large networks reveal that the dynamics tends to a natural stationary state called *balanced state*. In this state, a balance between the excitatory and inhibitory inputs emerges dynamically for a wide range of parameters, and the internal synaptic inputs act as a strong negative feedback, which linearizes the population responses to the external drive despite the strong non-linearity of the individual cells. This feedback also greatly stabilizes the system's state and enables it to track a time-dependent input on time scales much shorter than the time constant of a single cell (van Vreeswijk and Sompolinsky, 1998). Hence a balanced network configuration not only stabilizes, linearizes (and makes deterministic) the input-output transfer function, but also makes the network capable of fast tracking of temporal changes in the input.

It is worth noting that in a balanced network configuration a linear behavior emerges from the chaotic behavior of individual neurons, so that chaotic balanced networks can precisely track any input signals, and the tracked signals can be read out by averaging spikes over the whole network population (van Vreeswijk and Sompolinsky, 1996). Therefore, at a system level (i.e., considering large brain region networks) if a CNS response elicitation is considered as an input, the further network processing can be considered linear. Nonetheless the relation between an external energy flux and the corresponding CNS neuronal response is generally non-linear (e.g., an acoustic stimulation). This means that linear modeling techniques and methods can be adopted provided that the considered system input is represented by a given CNS neuronal population activity (see Equation 1), and, the (non-linear) mapping between an external stimulation and the corresponding CNS response has to be further derived when needed, understood as a separate issue.

#### 2.3.2. Neurophysiological *integration property* of active and reactive contributions

In this subsection we review empirical evidence about the property of the brain of integration of active and reactive contributions. In practice we argue that the overall response induced within the CNS is always determined by an active (energy based) and a reactive (pure information) contribution, and that the brain cannot discriminate between these two quantities during the response (or outcome) evaluation. This fact could also represent the basis of the placebo induced response (Puviani and Rama 2016).

Experimental verification of the influence of nonconscious conditioned stimuli on placebo/nocebo effects (Jensen et al., 2012, 2015) show that a reactive stimulus is able to interfere with a given active stimulation (e.g., an active drug or a painful stimulation), by increasing or decreasing the effect of the active response. This suggests that common active and reactive response components can be additive or competing and, hence, both contribute to the determination of the overall elicited response within the CNS. This observation is supported by further experimental results (Roy et al., 2009; Wagner et al., 2009; Wiech and Tracey, 2009) which show that emotional reactive stimulations (e.g., the subliminal perception of emotional pictures or other reactive emotional stimulations) modulate pain perception. A further interesting result which supports this line of reasoning comes from experiments reported in Plassmann et al. (2008), which show, by functional MRI studies, how prices of wine bottles (which represent here a piece of information related to the outcome) can affect the experienced pleasantness of the wine intaking (UCS). Indeed, the experienced pleasantness (which represents here the UCR) is due to the integration of the active component, *x*, and the reactive (self-induced) response *i*_*R*_, which is due to information processing. From the above mentioned results emerges evidence that emotional responses are additive, and can energize or decrease an active stimulation if they share common neuronal populations. Moreover, this property is not limited to emotional responses, but it also holds for other neuronal populations belonging to the reactive system (see Section 2.1). Indeed, pharmacological conditioning experiments (Amanzio and Benedetti, 1999; Benedetti et al., 2011) show that the conditioning (reactive) contribution increases the base active pharmacological effect of a given drug, even in animals (Guo et al., 2010). The additive property of emotional responses which became attributed to a given common stimulus is termed *integration property*. Considering one single neuronal population, the integration property can be expressed as:

(2) $$\begin{array}{l} y=x+i_{R}, \end{array}$$

where *y* represents the experienced CNS response, *x* represents the active response contribution and *i*_*R*_ the reactive response.

#### 2.3.3. Relation between predicted and reactive response: the reactive stability theorem

As described in the previous sections, whenever a source of stimulation (UCS) elicitates a CNS response (UCR) in a given organism a prediction error is computed as the difference between expected (or predicted) and experienced (i.e., UCR) responses; furthermore such an error signal updates the predicted response (i.e., UCS revaluation). Denoting *y*_*predicted*_ and *y* the predicted and the experienced response respectively, the prediction error computation can be expressed as *e* = *y* − *y*_*predicted*_. Furthermore, whenever a source of stimulation is perceived by an organism a reactive emotional response has to be elicited (in particular, as shown in the previous sections this is performed by the amygdala). Considering one single component (i.e., a specific neuronal population) of the given reactive emotional response, what can be said about its intensity? Does the reactive response coincide with the expected (or predicted) response? The following theorem proves that the reactive response associated with a given UCS has to be a fraction (i.e., less than the unity) of the expected response, in order to assure the stability of the emotional system. *The emotional system is said to be stable with respect to a given stimulus if and only if the response elicited by the stimulus does not increase unlimitedly over time*.

**Theorem**: *Necessary condition for the stability of the emotional system is that the emotional response associated to a given UCS is a fraction of the expected (predicted) response*.

The demonstration of the above theorem is provided in “Supplementary Data.”

On the basis of the Reactive Stability Theorem and of the reactive mimicking property, the reactive response of the generic neuronal population can be written as:

(3) $$\begin{array}{l} i_{R}=\alpha \cdot y_{predicted}, \end{array}$$

where the term α represents the intensity fraction (or *gain*) of the generic mimicked component belonging to the emotional system (such that |α| < 1). Generally speaking, if *K* represents the number of neural populations involved in the emotional response, a vector of K different values for the reactive gain α exists, in which every component is associated to a single neuronal population. The generic term α is also termed *emotional learning rate* in the following.

#### 2.3.4. Emotional contrast effects

In the technical literature it is well documented (Flaherty, 1982; Papini and Dudley, 1997) that surprising reward omissions, that is, the absence or reduction of an expected reward, are accompanied by aversive emotional reactions. On the other hand, surprising increases in the expected reward result in an appetitive emotional reaction. In particular, positive and negative *contrast effects*, arising from unexpected shifts in the obtained reward (whose value is greater or smaller than that previously experienced), depend on the comparison of the sensory property of the present stimulus with information stored in memory (Genn et al., 2004) and lead to an emotional response overshoot or undershoot, which is independent from the absolute value of the real reward. For instance, in Genn et al. (2004) it is shown that rats, in the presence of a shift from 32% to a 4% of the administered sucrose solution, displayed a successive negative contrast (i.e., a *depression effect* Flaherty, 1982) by initiating significantly fewer bouts of licking than control rats maintained on 4% sucrose. Furthermore, no significant increase in the dopamine efflux in the NAcc was observed during the consumption of 4% sucrose by rats that experienced the shift from 32%; on the contrary, the consumption of 4% sucrose by control rats was accompanied by a significant increase in the DA efflux in the NAcc.

The notion that contrast effects can be interpreted in terms of emotional responses is indirectly suggested by the effects of drugs on contrast (Flaherty, 1982). Indeed, experimental data reveal that drugs having anxiolytic effects on humans (e.g., amobarbital, ethanol, and benzodiazepines) tend to reduce negative contrasts. Furthermore, experimental results reviewed in Flaherty (1982) show an increase of the release of adrenocorticosteroid hormones in the presence of negative contrasts; this proves that a negative contrast is able to activate a component of the sympathetic response to stress, which, in turn, determine an emotional response.

Experimental evidence also shows that contrast exhibits an inverse dependence on the *inter trial interval*, denoted *T*, (i.e., the time interval between two successive stimulation trials) and a direct dependence with the magnitude difference between the preshift and the postshift values. The inter trial interval dependence suggests that modeling this effect should involve continuous time scale evaluations.

On the basis of the above mentioned results it is clear as emotional contrast effects have to be quantitatively taken into account in the model of the emotional response dynamics, since a difference between expected and actual stimulation determines inevitably an adding quantity in the final CNS response.

### 2.4. Model assumptions and hypothesis

In this Section the main hypothesis and assumptions adopted for the model development are summarized, on the basis of the results reviewed on the previous Sections.

H1 - *Definitions*: the definitions illustrated in Section 2.1 are adopted.

H2 - *Linearity hypothesis*: it is assumed that, at a *system level*, the linearity hypothesis holds for error signals and responses.

H3 - *Single emotional component*: we focus on the dynamics of a *single component* to ease the reading. This choice, however, does not entail any loss of generality, since our model can be applied to any component of the emotional system.

H4 - *Integration property*: as illustrated in Section 2.3.2 reactive and active responses add up as in Equation (2).

H5 - *Functional connectivity*: the structure of the dynamical emotional system (both in the discrete and in the continuous time scales) is expressed in Figure 1.

H6 - *Learning mechanisms*: it is assumed that both type of learning (CC and UCS revaluation) can co-occur simultaneously, and they are subjected to the constraints derived in Section 2.2 (see Figure 2).

H7 - *Prediction error computation*: prediction errors are computed as the difference between the expected/predicted and the experienced responses; furthermore, we will consider two different hypothesis for the expected response: (a) it coincides with the experienced response in the last trial, (b) it is computed as a filtered version (i.e., a weighted moving average) of the last trials outcomes.

H8 - *Stability of the emotional system property* holds (see Section 2.3.3 and Equation 3).

H9 - *Source Attribution*: it is assumed only one eliciting UCS and that it is correctly attributed by the emotional system. When a different scenario has to be considered it will be specified.

H10 - *Emotional contrast effects*: negative and positive contrast effects are evaluated as a linear function of the discrepancy between the expected and the incoming outcome.

H11 - *Discrete trials* (valid in the discrete time scale) - Multiple trials in the interaction between a source and a subject are considered; the trial duration Δ*T* is assumed to be relatively small and, in particular, negligible with respect to the *inter-trial interval* (ITI) *T*. For this reason, each trial can be ideally associated with a specific point on the time axis and the corresponding emotional response can be deemed constant.

H12 - *Residual response from previous trials* (valid in the discrete scale) - The time constant τ associated with the decay of the response elicited during each trial is deemed negligible respect to the inter-trial interval *T*; consequently, when a new trial takes place, the emotional response due to the previous trials has already vanished.

H13 - *Stimulus (UCS) perception* - It is assumed that the perceived UCS is the same in each trial, so as the associated contextual information and boundary conditions. This assumption states that, if a stimulus elicits a subject during the first trial in a specific context (e.g., place, timing, and specific boundary conditions), it has to be considered that the *stimulus perception* in the following trials involves exactly the same contextual and boundary conditions. In absence of such an assumption the reactive response elicited by the stimulus perception might be modulated by the different contextual information and boundary conditions. For instance, if an UCS is represented by a given drug, which has been encoded as UCS because previous interactions, then “UCS perception” refers to the UCS intake (in order to satisfy the same conditions occurred during the previous UCS-subject interactions), so that the reactive response associated with such a UCS can be triggered (independently from that the active pharmacological treatment has been altered).

H14 - *Recurrent patterns of stimulation* - A source of stimulation can elicit an organism with some regularities over time (or over discrete trials). For instance an electric shock device could stimulate a subject performing a periodic intensity pattern over time, or a given drug can exert a specific pattern of active effect over time (e.g., pharmacodynamic curve-related effects).

### 2.5. Model development

#### 2.5.1. Discrete time UCS revaluation model (without conditioning)

**Motivation:** the model accounts for a given UCS eliciting an organism with a variable active stimulation (*x*) and/or a variable reactive stimulation (*i*_*R*_).
**Hypothesis**: H1-5, H7a, H8-9, H11-13.

The discrete model is obtained through a *thought experiment* in which a given subject is stimulated by an UCS over successive trials. More specifically, the target UCS is perceived by the subject at every trial, in order to induce a reactive stimulation, after that an active UCS stimulation follows (e.g., through an electric shock delivery).

Provided that multiple stimulation trials are considered, it can be assumed that in every trial the expected (or predicted) response associated with the given UCS coincides with the last experienced outcome (which, in turn, coincides with the response experienced in the previous trial, H7a), or, alternatively, that the predicted response converges over successive trials to the actual experienced outcome (H7b). Without any loss of the generality, and with a first order approximation, it can be assumed that the predicted outcome is equal to the last experienced outcome. The expected response is updated through the prediction error computation over successive trials, in turn, the reactive response *i*_*R*_ will be updated according to Equation (3). In the first trial the reactive response is equal to zero, since the emotional system did not have any past learning experiences or interactions with the given UCS (so that the epxected response is zero); hence the elicited response is exclusively determined by the active stimulation:

(4) $$\begin{array}{l} y_{1}=x_{1}. \end{array}$$

Since the expected outcome was equal to zero for that UCS, the prediction error after the first active stimulation trial is equal to *x*_1_ and, such an error updates the expected response for the new trial, and, consequently the reactive response, according to Equation (3), that is:

(5) $$\begin{array}{l} y_{expected,\;2}=x_{1} \end{array}$$

(6) $$\begin{array}{l} i_{R2}=\alpha \cdot x_{1}. \end{array}$$

In the second trial, as soon as the UCS is perceived the learned reactive response will be triggered, which, together with the active stimulation determine the CNS response (see Equation 2):

(7) $$\begin{array}{l} y_{2}=x_{2}+\alpha x_{1}; \end{array}$$

moreover, a new error signal is computed as

(8) $$\begin{array}{l} e_{2}=y_{2}-y_{1}=x_{2}+\alpha x_{1}-x_{1}. \end{array}$$

Without loss of the generality, it can be assumed that the active elicitation is kept constant at every trial (i.e., *x*_*n*_ = *x*∀*n*, where *n* represent the trial index), so that the error at the second trial can be expressed as

(9) $$\begin{array}{l} e_{2}=\alpha \cdot x \end{array}$$

and the reactive response updated at the end of the second trial is given by

(10) $$\begin{array}{l} i_{R3}=\alpha x+\alpha^{2}x. \end{array}$$

It easy to demonstrate that the response elicited in the *n*-th trial can be expressed as:

(11) $$\begin{array}{l} \begin{array}{c} y_{n}=x_{n}+\alpha \cdot \sum_{k=1}^{n-1}e_{k}=\\ x_{n}+\alpha \cdot \sum_{k=1}^{n-1}\left(y_{k}-y_{k-1}\right), \end{array} \end{array}$$

which, can be reformulated, as:

(12) $$\begin{array}{l} y_{n}=x_{n}+\alpha \cdot y_{n-1}. \end{array}$$

The last equation shows that the overall CNS response is determined by the contributions of the active elicitation (*x*_*n*_) and of the reactive (emotional) contribution, determined by previous learning, and expressed as a fraction of the expected outcome, which, it has to remember, it is assumed to coincides with the response elicitation in the previous trial, with a first order approximation.

If a constant active elicitation *x* is considered over successive trials, it is easy to show that the response approaches the asymptotic value

(13) $$\begin{array}{l} y_{\infty }=\frac{x}{1-\alpha } \end{array}$$

as *n* increases.

When the asymptotic value has been reached, the prediction error will be zero for every successive stimulation trials, and no predicted response updating can occur. The error signal will be zero also if the condition *y*_*n*_ = *y*_*n*−1_ occurs in the generic *n*-th trial. As will be shown in the Section “Results,” some psychophysiological phenomena (e.g., evaluative conditioning) and neuropsychiatric pathologies (e.g., PTSD) occur if the above mentioned condition holds (together with the following conditions: (a) the reactive response is different from zero and (b) the active response is zero. Both conditions can be expressed in terms of the following: the expected or predicted response coincides with the reactive/self induced response). Furthermore, it is easy to prove that if a series of successive trials in which the active stimulation (*x*) is kept equal to zero, the CNS response in the *n*-th trial can be expressed as:

(14) $$\begin{array}{l} y_{n}=\alpha \cdot y_{n-1}=y_{0}\alpha^{n}, \end{array}$$

where *y*_0_ represents the expected response before the beginning of the UCS devaluation process. Hence, during devaluation, the response tends asymptotically to zero.

**Contrast Effects**: hypothesis H10 is added in the model.

Contrast effects can be included in the discrete model of implicit emotional learning by adding a new function, called *contrast function* and denoted *C*_*eA*_, which, generally speaking, could be a function of the *actual error-signal* (denoted *e*_*A*_), defined as

(15) $$\begin{array}{l} e_{A,n}≜\left(x_{n}+\alpha \cdot y_{n-1}\right)-y_{n-1} \end{array}$$

for the *n*-th trial; note that this definition is motivated by the fact that the actual error signal refers to the actual trial (instead of the previous one), since contrast effects occur in parallel with the actual outcome. Hence, the emotional response in the *n*-th trial can be expressed as (see also Equation 12)

(16) $$\begin{array}{l} y_{n}=x_{n}+\alpha \cdot y_{n-1}+C_{eA}\cdot e_{A,n} \end{array}$$

if *e*_*A, n*_ ≠ 0 and

(17) $$\begin{array}{l} y_{n}=y_{n-1} \end{array}$$

if *e*_*n*_ = 0 and *e*_*A, n*_ = 0.

Assuming a simple linear contrast function *C*_*eA*_ ≃ *K* and assuming 0 < *K* < 1 (for emotional stability reasons), it is easy to demonstrate that Equation 12 becomes

(18) $$\begin{array}{l} y_{n}=\left(1+K\right)\cdot x_{n}+\left(\alpha +K\alpha -K\right)\cdot y_{n-1}. \end{array}$$

On the basis of the above reported results, if an unexpected UCS active elicitation occurs (i.e., an active UCS stimulation which is not signaled by any CS nor by a prior UCS perception; for instance, this scenario can be represented by a laboratory setting where a permanently-connected electric shock device elicits a subject without any prior signaling), it determines the response

(19) $$\begin{array}{l} y_{UCS}=x+K\cdot x, \end{array}$$

and is attributed to the UCS. Moreover, a prediction error is computed and the reactive response associated with the UCS is updated; more specifically, if the expected response before the unexpected elicitation was equal to *x* + α*x*, the prediction error is computed as *e* = *x* · (*K* − α). Furthermore, if another unexpected UCS elicitation occurs, the resulting prediction error is equal to zero since the expected outcome is now equal to the actually experienced outcome, which is given by *x* + *K* · *x* (i.e., is determined by the active elicitation *x* and the contrast contribution due to the unexpected elicitation *Kx*). This mathematical result shows that a series of trials of unexpected UCS elicitations lead to a computation of a prediction error only in the first unexpected elicitation; indeed, in the successive unexpected trials only a constant reactive contribution (i.e., *Kx*) due to the contrast effect is elicited. Indeed, if at every unexpected UCS stimulation an error signal was computed, then the UCS revaluation would lead to an unbounded increase of the UCS expected response, which actually is not the case. The above mentioned results and observations lead to a novel interpretation of experimental results (Schultz et al., 1997; Hollerman and Schultz, 1998; Schultz, 2002) in relation to the recording of the activity of dopamine neurons in the VTA during unexpected rewarding stimulations (See Section 3.2.4). Hence, unexpected stimulations represent a particular case of emotional contrast effect, in which the expected response was zero; furthermore, since no error signal is computed, such an increase in dopamine response due to unexpected stimulation simply reflect a reactive response, which, in turn, may subserve as an incentive to orienting and focus attention on the source of stimulation and on eventual suspicious statistical or contextual contingencies (in other words, in this case the dopamine response is not computed to update the UCS value, but for focus attention in order to observe if some contingent cues with the unexpected UCS release occurred). It is worth noting that the same situation occurs also when an expected reward is omitted (i.e., negative contrast effect), in this case the induced negative reactive response does not update UCS values, instead it focuses attention to discover eventual contingent cues (such cues, if do exist, can become *conditioned inhibitors*; Harris et al., 2014). As will be shown in Section 3.2.4 dopamine neurons in the VTA and substantia nigra can subserve to other brain regions (e.g., the OFC) to compute both error signals and reactive responses (associated to UCSs, or due to contrast effects in order to focus attention and facilitate further learning).

**Hypothesis, H7b**: the expected response is computed as an exponential weighted average of the last trials outcomes. This hypothesis is motivated by the consideration that the expected/predicted responses can be shaped considering different previous outcomes and not only the last one. This hypothesis is confirmed by experimental results (Bayer and Glimcher, 2005) which show that dopamine neurons *encode the difference between the current reward and an exponentially weighted average of previous rewards*.

Under the H7b hypothesis, the predicted response, denoted < *y*_*n*−1_ > (since it represents the filtering function of the last responses until that occurred in the n-1 trial), can be expressed as:

(20) $$\begin{array}{l} <y_{n-1}>=\overset{L}{\underset{k=1}{\sum }}h_{k}\cdot y_{n-k}, \end{array}$$

where *L* represents the number of the responses involved in the filtering process, and *h*_*k*_ represents the generic k-th filter coefficient (or weight). Note that Equation (20) expresses a general moving filtering function and not only an exponential one. It can be shown that a moving exponentially averaged filter (in discrete time) represents a *low pass filter* (and also that its continuous-time counterpart is an R-C first order type filter; Schafer and Oppenheim, 2009). Substituting Equation (20) in Equation (12) yields:

(21) $$\begin{array}{l} y_{n}=x_{n}+\alpha \cdot \overset{L}{\underset{k=1}{\sum }}h_{k}\cdot y_{n-k}. \end{array}$$

Furthermore, considering that the error signal is now computed as:

(22) $$\begin{array}{l} e_{k}=y_{k}-<y_{k-1}>, \end{array}$$

Equation (21) can also be written as (see also the first line of Equation 11):

(23) $$\begin{array}{l} y_{n}=x_{n}+\alpha \cdot \overset{n-1}{\underset{k=1}{\sum }}\left(y_{k}-\overset{L}{\underset{v=1}{\sum }}h_{v}\cdot y_{n-v}\right). \end{array}$$

**Adding Hypothesis, H14**: the active stimulation presents a recurrent pattern over successive trials (e.g., a gaussian shaped curve for the intensity of the stimulation occurs periodically).

If the brain recognizes recurrent patterns of stimulation over time (i.e., a typical stimulation intensity pattern), such an information will be exploited for a more precise inference of the probable outcome. This line of reasoning is supported by experimental results which show that neurons encode precise timings between stimuli (Schultz et al., 1997); for instance, dopamine neurons learn, after few observations, that after a prescribed time from the onset of a cue a given quantity of reward will be delivered.

Learning statistical regularities and patterns represent a type of CC learning, since the variable “time” and “temporal relations” can be considered as contextual cues (Bouton, 1993). For this reason we speculate that the pattern recognition function may be performed by the hippocampus, together with OFC interactions for the inference of more complex patterns. In practice, what is learned in this case is that at a given “time reference” (CS1) a specific UCS intensity stimulation occurs, then at a successive time reference (CS2) a different UCS intensity stimulation occurs and so on, until an eventual entire recurrent pattern of stimulation will be learned. From a modeling perspective it is important to note that if the brain “is sure” about the fact that a specific pattern of stimulation is occurring (denoted *y*_*P*(1..*N*)_ where the interval (1..*N*) represents the entire range of trials the pattern comprises), then the predicted response at the generic *n*-th trial is represented by the corresponding intensity within the pattern (i.e., *y*_*Pn*_). Conversely, if the brain does not recognize any pattern, no adding inference can be performed and the predicted response is computed as in Equation (20). Nevertheless, intermediate situations between the above mentioned extremes can occur; more precisely during pattern learning, or whenever the recognized probability of having a given pattern is not equal to one, the expected response has to be computed as a combination of the actual UCS revaluation contribution (Equation 20) and of the response expected by the pattern. It is easy to prove that the predicted (expected) response can be expressed as:

(24) $$\begin{array}{l} <y_{n-1}>=\omega_{NP}^{\left(n\right)}\cdot \overset{L}{\underset{k=1}{\sum }}h_{k}\cdot y_{n-k}+\omega_{P}^{\left(n\right)}\cdot y_{Pn}, \end{array}$$

where $\omega_{P}^{\left(n\right)}$ ($\omega_{NP}^{\left(n\right)}$) represent the weight (or *belief confidence*) related to the occurrence (no occurrence) of the given pattern at the *n*-th trial; furthermore, it has to be satisfied the following equality:

(25) $$\begin{array}{l} \omega_{NP}^{\left(n\right)}=1-\omega_{P}^{\left(n\right)}. \end{array}$$

Equation (24) shows that the predicted response comprises two contributions: (a) the UCS revaluation component, which represents the *bottom-up contribution*, since it is determined by the actual perception of the response (or its gradient over time) and it is exploited from higher level neuronal networks to form more complex hierarchical patterns; (b) the contribution due to previous inferential learning, which represents the *top-down contribution*, since it is encoded by higher level hierarchical neural structures and exploited to compute a reactive response which will be perceived by lower level structures for the computation of the prediction error.

In order to include the contrast effect in the model an additional term proportional to the *actual error signal* has to be considered. It is easy to show that the actual error signal can be computed as:

(26) $$\begin{array}{l} e_{A,n}=x_{n}+\left(\omega_{NP}^{\left(n\right)}\overset{L}{\underset{k=1}{\sum }}h_{k}\cdot y_{n-k}+\omega_{P}^{\left(n\right)}y_{Pn}\right)\cdot \left(\alpha -1\right). \end{array}$$

**Figure 3:**
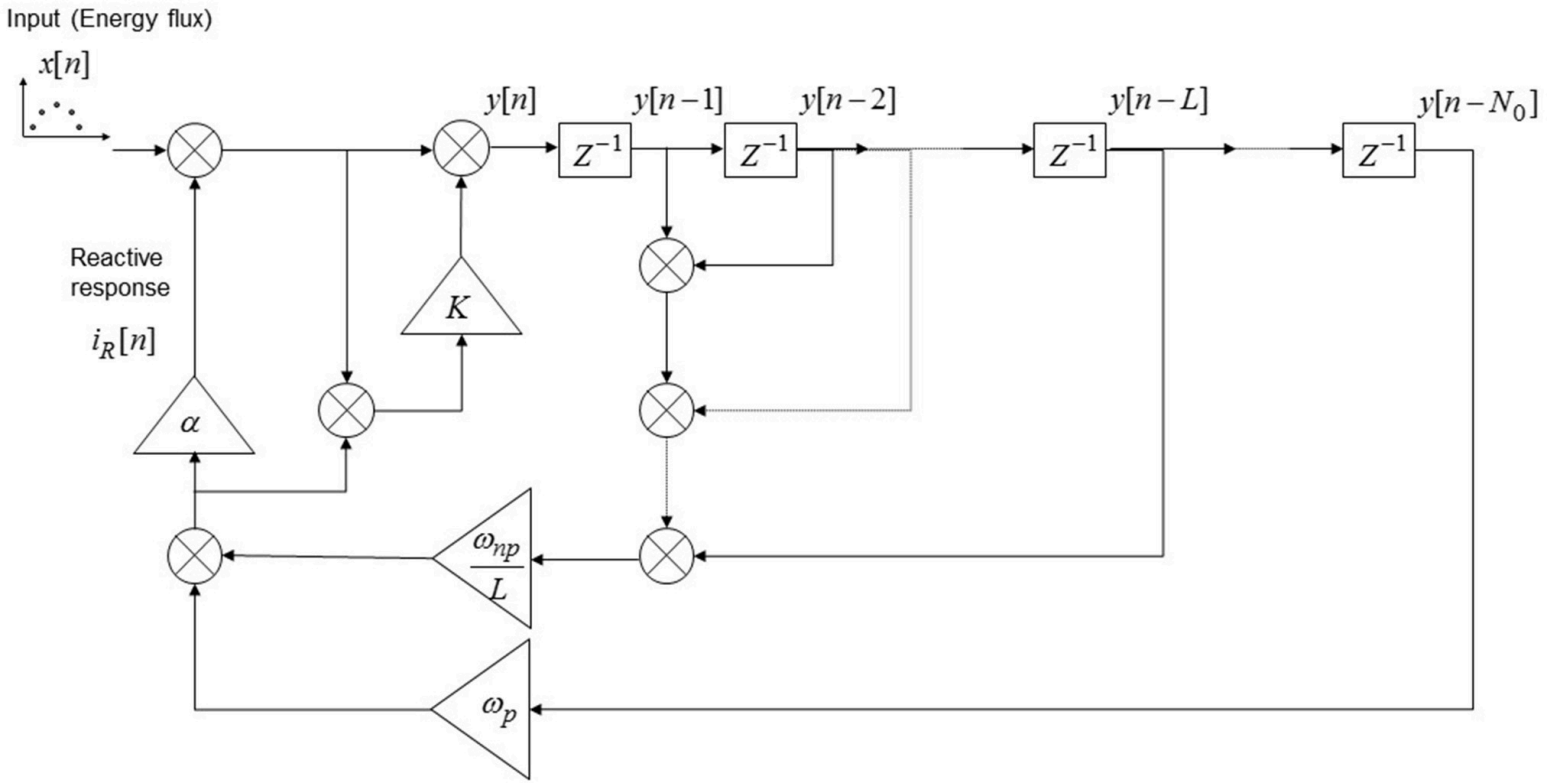
**Discrete-time model implementation of the emotional system dynamics**. The input to the system (*x*[*n*]) is represented by a series of discrete stimulations over successive trials; the emotional response in the n-th trial is given by *i*_*R*_[*n*] and the overall CNS response is given by *y*[*n*]. The blocks “*Z*^−1^” represents one unit delay, the triangular blocks represent multiplicative factors and all the nodes are summation nodes. The model takes into account of the implicit UCS revaluation, of the contrast effect and of the “pattern recognition” in the case of a periodical stimulation pattern of period *N*_0_.

Hence, the discrete emotional learning model of a periodic stimulation of period *N*_0_ (i.e., such that *y_n_* = *y*_*n*−*N*_0__) can be written as:

(27) $$\begin{array}{l} y_{n}=x_{n}+\alpha \omega_{NP}^{\left(n\right)}\overset{L}{\underset{k=1}{\sum }}h_{k}\cdot y_{n-k}+\alpha \omega_{p}^{\left(n\right)}y_{n-N_{0}}+K\cdot e_{A,n} \end{array}$$

and its implementation is reported in Figure 3.

#### 2.5.2. Discrete time classical conditioning model with implicit UCS revaluation

**Motivation:** derivation of a discrete model for CC under the stochastic Hebbian plasticity hypothesis and considering the implicit UCS revaluation during acquisition process.
**Hypothesis**: H1-6, H7a, H8-13.

In this Section a discrete model of classical conditioning which accounts for the implicit UCS revaluation is presented, and its derivation is developed through a thought experiment, where a sequence of trials, involving CS-UCS-subject interactions, is analyzed. Our derivation relies on the assumption that the CS-UCS synaptic connections are governed by the mechanisms of *stochastic Hebbian plasticity* (Hebb, 1949; Amit and Fusi, 1994; Fusi, 2002; Soltani and Wang, 2006, 2010; Fusi and Abbott, 2007). This hypothesis is supported by both some experimental results shown in Redondo et al. (2014) and Gore et al. (2015), and other models relying on the fact that a CS-UCS pairing entails the Hebbian potentiation of the CS inputs onto the UCS representations in the BLA (Sah et al., 2003; Pickens and Holland, 2004; Pape and Pare, 2010). Hebbian learning is based on the idea that synapses between neurons being simultaneously active become stronger. Consequently, “neurons that fire together wire together” through an increase in synaptic efficacy mediated by *long term potentiation* (LTP); on the other hand, a decrease in synaptic efficacy is mediated by *long term depression* (LTD). The mathematical derivation of the proposed model is available in “Supplementary Material” Section.

The mathematical analysis results in the following model for classical conditioning in the discrete time scale:

(28) $$\begin{array}{l} \omega_{CS-UCS}^{\left(n\right)}=\omega_{CS-UCS}^{\left(n-1\right)}+{\hat{\alpha }}_{+}\cdot \left(1-\omega_{CS-UCS}^{\left(n-1\right)}\right) \end{array}$$

(29) $$\begin{array}{l} \omega_{CS-UCS}^{\left(n\right)}=\omega_{CS-UCS}^{\left(n-1\right)}-{\hat{\alpha }}_{-}\cdot \omega_{CS-UCS}^{\left(n-1\right)} \end{array}$$

(30) $$\begin{array}{l} i_{R}^{\left(n\right)}=\alpha \cdot \left(X+i_{R}^{\left(n-1\right)}\cdot \omega_{CS-UCS}^{\left(n\right)}\right) \end{array}$$

(31) $$\begin{array}{l} y_{CS}^{\left(n\right)}=\omega_{CS-UCS}^{\left(n\right)}\cdot i_{R}^{\left(n-1\right)}. \end{array}$$

Note that Equations (28) and (31) hold for *n* ≥ 2 and that the initial conditions

(32) $$\begin{array}{l} \omega^{\left(1\right)}=0 \end{array}$$

(33) $$\begin{array}{l} i_{R}^{\left(1\right)}=\alpha \cdot X \end{array}$$

(34) $$\begin{array}{l} y_{CS}^{\left(1\right)}=0 \end{array}$$

and

(35) $$\begin{array}{l} y_{UCS}^{\left(1\right)}=X \end{array}$$

should be adopted when employing them. The term $\omega_{CS-UCS}^{\left(n\right)}$ is termed *synaptic strength* and represents the fraction of synapses from the neurons representing the CS stimulus onto the encoding neurons for the UCS in the *n*-th trial; the terms ${\hat{\alpha }}_{+}$ and ${\hat{\alpha }}_{-}$ in Equations (28 and 29) represent the *potentiation* and the *depression* rates respectively, and they determine the probability for plastic synapses to switch from the depressed to the potentiated state and vice-versa. If UCS revaluation during conditioning is neglected the proposed model coincides with the well known Rescorla-Wagner (R-W) model (Miller et al., 1995). Our extended model provides a more general and accurate description of the emotional response during conditioning than the original R-W model for different reasons which are described in Section 3.2.4.

#### 2.5.3. Conditioning to a reactive source stimulus

Three possible events can occur when a CS is conditioned to a purely reactive UCS (i.e., an UCS for which no active component elicitation is expected, e.g., an emotional picture): (1) a simple associative connection CS-UCS is generated; (2) the CS is misattributed to be the source of the elicited response, so becoming a new (and independent) reactive source like the original UCS, so that a new reactive response, equal (but independent) to the original *i*_*R*_, is generated and associated to the CS. Furthermore, this misattribution process could occur even during the presentation of the CS alone after conditioning, since the reactive elicitation (which is equal to ω_*CS*−*UCS*_ · *i*_*R*_) could be misattributed forward the CS, in this case the reactive response being associated with CS corresponds to the quantity ω_*CS*−*UCS*_ · *i*_*R*_. (3) A combination of the previous two events could occur. Moreover, if one of the two last mentioned events occurs, the conditioned CS may become an inextinguishable element of emotional reaction, since the expected response is purely reactive and coincides with the elicited reactive response (i.e., the prediction error is always zero).

### 2.6. Emotional system dynamics in continuous time scale

In the previous Sections a discrete-time model for the computation of the emotional response in different scenarios has been developed. In real world conditions, however, a stimulation might elicit continuously a subject. From a modeling perspective a continuous elicitation can be seen as a series of an infinite number of discrete trial stimulations, each of which has an infinitesimal time duration and the temporal spacing between them tends to zero. In these conditions the emotional response is continuously updated driven by the continuous time counterpart of the prediction error. In the following, the problem of developing a system computational model for describing the continuous time evolution of the emotional response is investigated. More specifically, our approach is based on standard engineering methods. Without any loss of the generality we focus here on the development of the model in absence of the pattern recognition contribution, since we focus here on the dynamics of the emotional control system; furthermore, such a feature can be modeled by a neural network for pattern recognition which operates in parallel with the OFC.

In order to obtain a continuous counterpart of a discrete model the *sampling time* has to be known. Generally speaking the sampling time is defined as the time period at which the continuous time model is sampled to obtain a discrete version of it. In our analysis we started developing directly a discrete model, since the experimental results available in the literature are based on discrete trials measures. For this reason we have now to infer the continuous model whose sampling would produce the discrete model obtained in the previous sections. Considering that the discrete model holds for any arbitrary large inter-trial interval (denoted *T*) and that we are interested in finding the emotional dynamics in the limit of *T* which tends to zero, it is possible to consider the smallest *T* such that the discrete model holds, and then assuming it as the sampling time. The *sensory time discrimination threshold* (Luna et al., 2005) represents the smallest temporal interval for which the CNS neurons can discriminate between two distinct consecutive stimulations, hence, this parameter is taken as the sampling time (*T*). It is worth pointing out that the value of *T* depends on the involved perceptive modality (e.g., somatosensory stimulation, visual stimulation or acoustic stimulation), and that, if an active stimulation varies faster than *T* the neurons encode an average value within a time window of *T*. For instance, providing that in the visual stimulation *T* is about 100 ms, the stimuli variation within 100 ms are encoded as a mean value over 100 ms.

Assuming the discrete model can be obtained sampling the continuous time counterpart of it with a sampling time *T*, the continuous model can be obtained through the following procedure:

(a) the *transfer function* of the discrete recursive difference system in the Z-domain (Schafer and Oppenheim, 2009) is computed; (b) the transfer function of the continuous dynamic system is obtained in the *S*-Laplace domain substituting the Z variable of the discrete transfer function with the following equation:

(36) $$\begin{array}{l} Z=\frac{1+s\cdot T∕2}{1-s\cdot T∕2} \end{array}$$

where the variable *s* represents the Laplace variable (this substitution represents the inverse operation of the so called *bilinear transform*; Schafer and Oppenheim, 2009); (c) if needed, the differential equation in the time domain (or the continuous time state space representation) is obtained with the inverse Laplace Transform of the equation in the Laplace domain obtained in the previous stage b.

**Figure 4:**
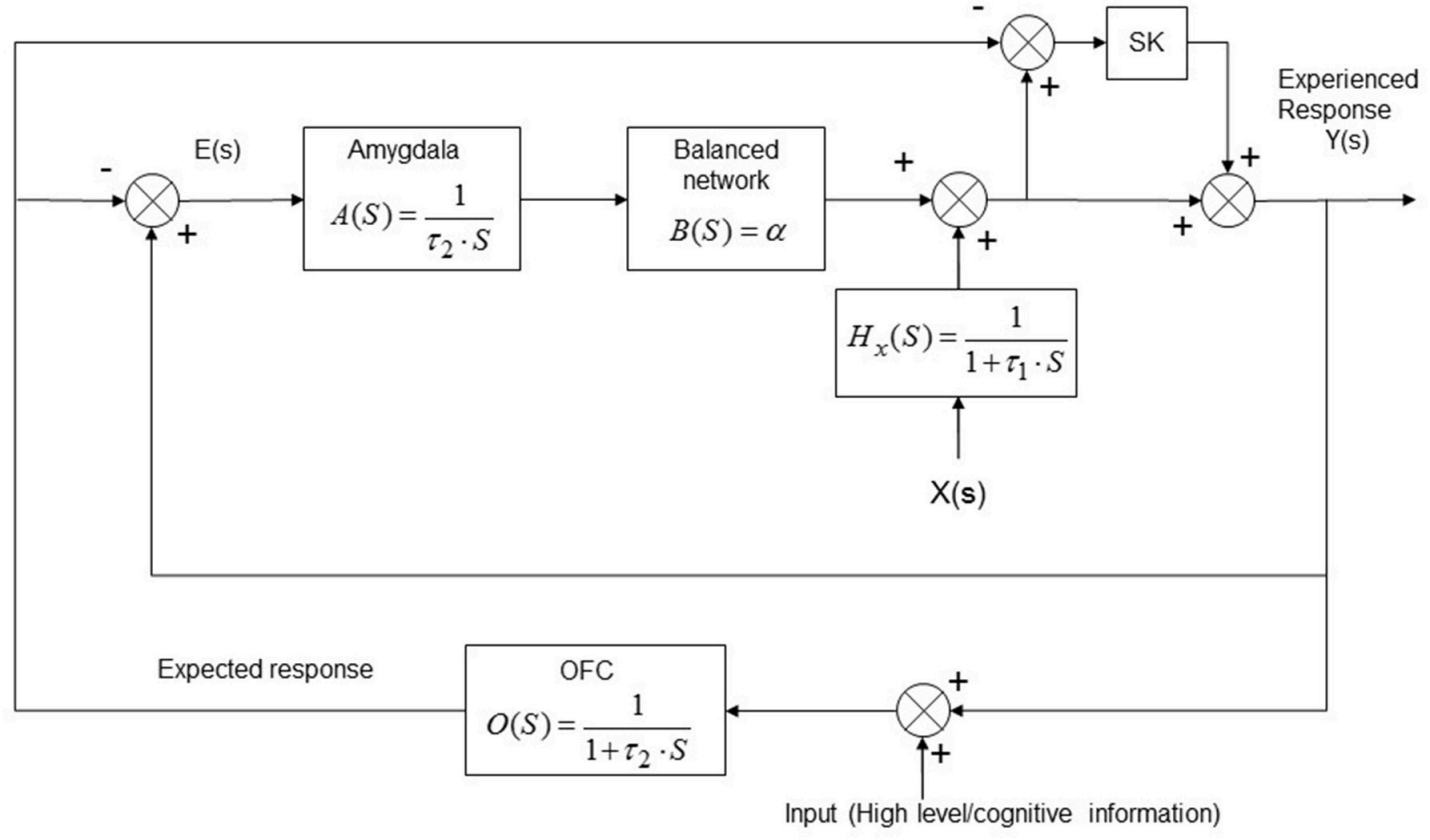
**Continuous-time model implementation of the emotional system**. The model is presented in the Laplace domain where “S” represents the Laplace operator. The functional connections between the involved actors and the processing task accomplished by each block are shown. The model takes into account of the implicit UCS revaluation and of the contrast effect. X(s) represents the input (energy flux), Y(s) the CNS response and E(s) represents the prediction error; αis the emotional learning rate for the neuronal target population, K is the contrast effect parameter. OFC, orbitofrontal cortex.

Applying the aforementioned procedure to the discrete model (Equation 27) the continuous dynamic system can be developed and its *transfer function* in the Laplace domain can be expressed as:

(37) $$\begin{array}{l} H\left(s\right)=\frac{Y\left(s\right)}{X\left(s\right)}\\ =\frac{s^{2}+K\tau_{2}+s\left(K+\tau_{2}\right)+1}{s^{2}\tau_{1}\left(\tau_{2}+K-K\alpha \right)+s\left(\tau_{2}+K-K\alpha +\tau_{1}-\alpha \tau_{1}\right)+1-\alpha } \end{array}$$

where the term τ_1_ represents the time constant of the equivalent low pass filter relative to the *x*(*t*) neuronal population target (and it is closely related to the time discrimination threshold T); the term τ_2_ represents the equivalent time constant of the low pass filtering effect performed by the emotional evaluation system (i.e., the equivalent of the exponential weight moving average in the discrete time model); *Y*(*s*) and *X*(*s*) represent the overall response and the input (i.e., the active stimulation) in the Laplace domain respectively. Taking into consideration the constraints and the functional connections derived in the previous sections (see Figure 1), the implementation of the derived transfer function model can be obtained as depicted in Figure 4.

It is easy to prove that the system differential equation obtained from the continuous model represented in Figure 4 is:

(38) $$\begin{array}{l} \ddot{y}\left(t\right)\cdot \tau_{1}\left(\tau_{2}+K-K\alpha \right)+ẏ\left(t\right)\left(\tau_{2}+K-K\alpha +\tau_{1}-\alpha \tau_{1}\right)\\ +\;y\left(t\right)\left(1-\alpha \right)=ẍ\left(t\right)\cdot K\tau_{2}+ẋ\left(t\right)\left(K+\tau_{2}\right)+x\left(t\right) \end{array}$$

where the functions *y*(*t*), ẏ(*t*), $\ddot{y}$(*t*),*x*(*t*), ẋ(*t*) and ẍ(*t*) represent the elicited response, its first and second derivatives, the active stimulation and its first and second derivatives over time, respectively.

## 3. Results

In this Section some results from the theory and the developed models, understood as postpredictions and quantitative explanations of experimental observations reviewed from the technical literature, are presented. Successively a model comparison with existing models is provided. Furthermore, a section describing model validation, interpretation and applicability to some research topics is presented.

### 3.1. Summary of the derived models

All the models are based on the functional structure described in Figures 1, 2. The main assumptions are summarized in Section 2.4.

A simplified discrete model for UCS revaluation and contrast effects is described by Equation (18) and it is named M1 in the following.

The discrete model for UCS revaluation, contrast effects and pattern recognition (named M2) is expressed by Equation (27).

The discrete model for CC and implicit UCS revaluation (named M3) is expressed by Equations (28–35).

The continuous time system dynamical model (named M4) can be expressed by the transfer function in Equation (37) and represented in Figure 4, and by Equation (38).

### 3.2. Post-predictions and quantitative explanations

#### 3.2.1. Resistant-to-extinction responses

Generally speaking, on the basis of the theory and models derived in the previous sections, an emotional response can become resistant to extinction (or inextinguishable) if (1) the prediction error is zero while (2) the reactive response is different from zero while (3) the active response is zero; or, equivalently, if the reactive response coincides with the expected response. Indeed, since, in general, the emotional system tracks an active component, it is obvious that whenever such a component decrease or vanishes then the associated reactive response will be decreased too. However, in the continuous time scale, there are two cases which can lead to a situation in which the active stimulation drops to zero and the reactive response does not (so that it becomes inextinguishable): (a) if a saturation level of the expected response is reached; (b) in the presence of specific dynamics of the active stimulation, exploiting the inertial nature of the emotional tracking system. We will describe in detail the first case, and we propose future computational and experimental researches to investigate the second.

On the other hand, in the discrete time scale it is possible inducing resistant to extinction responses through the misattribution phenomena (see Section 2.2.6). In fact, if a purely reactive response is attributed (and hence expected) to a given stimulus, then it will induce the same reactive response which is expected (i.e., the prediction error is always zero). A quantitative analysis of such a case will be provided next.

#### 3.2.2. Inextinguishability through emotional response saturation and hippocampus impairment

During an extreme traumatic event different phenomena may occur, and, our model shows how these conditions can determine a resistant-to-extinction emotional reaction.

Generally speaking, a traumatic response is mainly determined by an automatic inferential emotional learning, instead that by an active energy-based stimulation *per se*. More specifically, the processing of traumatic information, such as an imminent death danger, can be implicitly processed by the OFC which, in turn, activates the amygdala through the computation of prediction errors. Indeed, experimental results from the literature (Steinberg et al., 2013; Sadacca et al., 2016) show that *inferred* outcomes (e.g., rewards), never directly experienced before, determine prediction errors (for instance computed in the VTA) which are just like predictions based upon direct experience/stimulation. In practice this means that the OFC can control the computation of error signals, which stimulate the amygdala, on the basis of the *difference of the actual expected outcome and the inferred outcome* (through information processing) associated with a given UCS or situation, even before experiencing a direct active (energy-based) stimulation. From a modeling perspective this situation can be described assuming that the *input* to the emotional system is the *error node* (see Figure 4), and that the prediction error is proportional to the difference between the inferred and the expected outcome. Indeed, experimental results based on optogenetic manipulations (Chang et al., 2016) have shown that inducing artificial prediction errors within the VTA permits to induce behaviors and responses like those obtained by inference and statistical learning. Moreover, it has been observed that prediction errors are transmitted in spikes-form (i.e., very short and relatively small decrease in neuron firing rates); we argue this modality represents a suitable method to send direct (inferred) error signals since the transfer function between the *error node* (E(s)) and the output (Y(s)) is unstable, so that a continuous error signal could rapidly lead to an unlimitedly increasing of the output (it is easy to prove that the Y(s)/E(s) transfer function represents an unstable system; see Figure 4).

**Figure 5:**
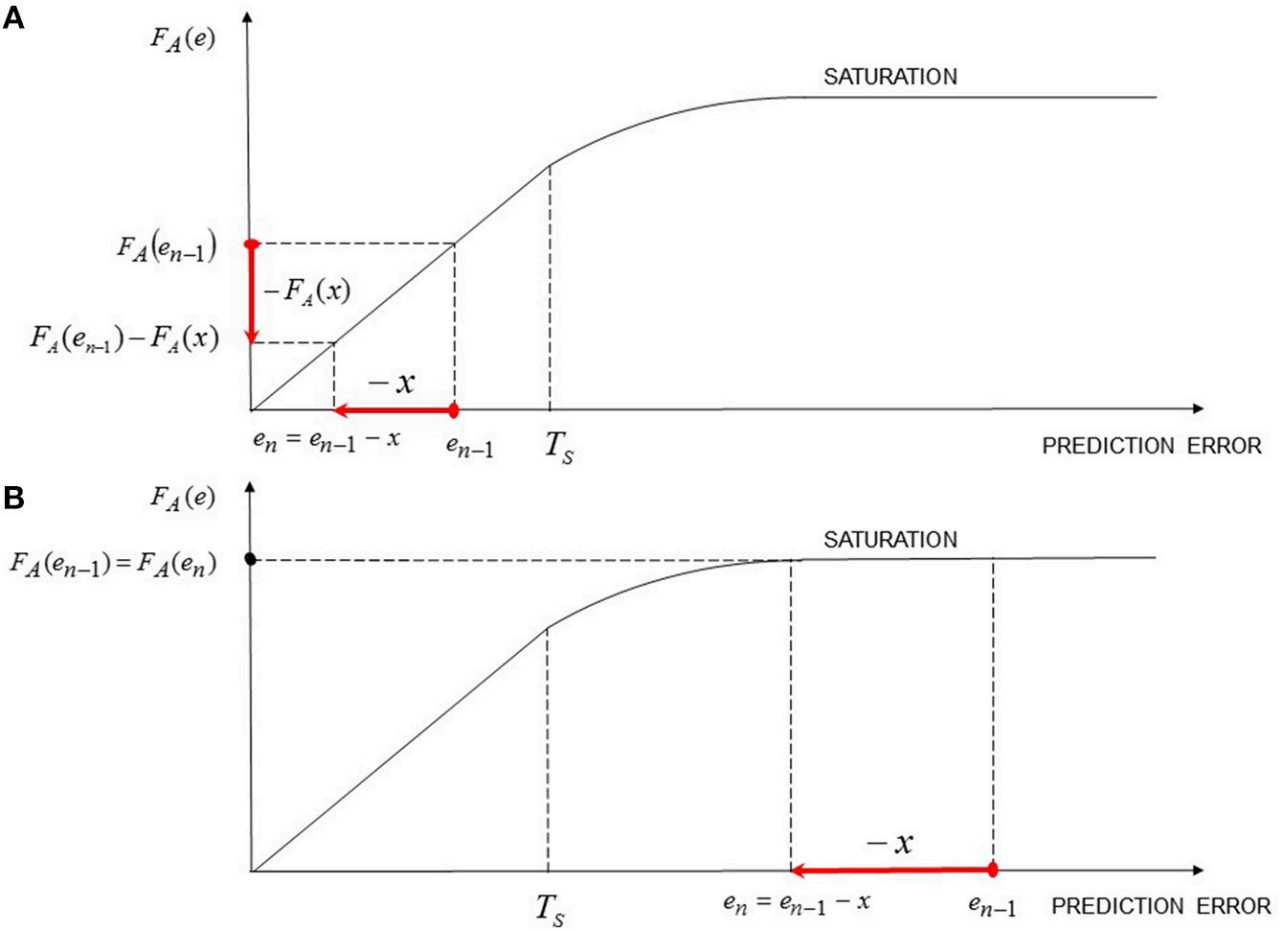
**Resistant-to-extinction emotional reaction through response saturation**. Schematic representation of the biological behavior of the amygdala function *F*_*A*_(*e*) in its linear zone **(A)** and in its saturation zone **(B)**. In case **(A)** the prediction error (equal to −*x*) is able to reduce the elicited emotional response. On the contrary, in case **(B)** a negative prediction errors unable to produce a similar effect, so that the emotional response remains at its saturation level. In particular, the case **(B)** occurs if the negative prediction error (due, for instance, to the fact that the active response *x* is no more elicited during the stimulation or because the stimulation is occurring in a safe context) is smaller than the degree of saturation reached by the amygdala in the previous stimulation(s).

Nevertheless, if the inferred outcome is relatively greater than the expected response encoded within the OFC (associated with the target situation or stimulation), then the inferred prediction error could become so intense to determine a sort of saturation of the amygdala response. Indeed, any mathematical function *f*(*x*) representing a specific *biological response* cannot take on arbitrarily large values, because of the limited dynamics of the response itself. In practice, as the value taken on by the argument *x* grows, the corresponding value *f*(*x*) of the function does not steadily increase in proportion to it and, when *x* crosses a certain threshold, a certain saturation level is reached; in other words, *f*(*x*) exhibits a *nonlinear* behavior for sufficiently large values of *x*. In particular, these considerations hold for the amygdala, which takes prediction errors as input and it updates the predicted outcome on the basis of such errors; we denoted such a behavior *F*_*A*_(*e*) = *Y*_*expected*_*(within the amygdala)*. Hence, the linearity hypothesis holds if in any trial (or at any specific time instant) the prediction error takes on a value smaller than a *saturation threshold T*_*S*_, which defines the linearization range for the amygdala expected response function (*F*_*A*_(*e*); see Figure 5). If, however, the prediction error exceeds *T*_*S*_ (i.e., if the error becomes excessively large), a phenomenon of *emotional saturation* should be expected. When emotional saturation occurs, the source of stimulation (or any associated cue) generating it could produce inextinguishable effects, even in the absence of any active stimulation or if the exposition to a trauma-related stimulus occurs in a *safe context*. In fact, if the prediction error computed because a safe context is smaller than the reached degree of saturation, then its inhibitory effect does not determine any response reduction(see Figure 5). Moreover, it is important to note that contextual information are primarily coded and stored in the hippocampus (while the representation of an aversive stimulus is coded within the BLA), so that, if during the traumatic event the hippocampus does not properly code contextual information, then no effective contextual discrimination can be obtained during further exposures of the stimulus. As far as this last point is concerned, it is worth mentioning that hippocampus functioning and its ability to encode contextual information are impaired by uncontrollable stress together with an hyperactivation of the amygdala, as shown in a model developed in Kim and Diamond (2002). Furthermore, if hippocampus activities are impaired during high stress exposure, it is likely that the amygdala associates every contextual cue directly (even if insignificant) with the eliciting reactive response (i.e., misattribution), so that, successively, they will be able to trigger the reactive response (Bechara et al., 1995). These considerations lead us to the conclusion that the traumatic emotional response becomes resistant-to-extinction since the expected response coincides with the reactive triggered response. We argue that this phenomenon could happen in panic disorders and PTSD (Beck and Sloan, 2012; Parsons and Ressler, 2013; Perusini et al., 2016). As a matter of fact, in some forms of PTSD and panic disorders the mere repetitive exposure to cues related to a traumatic event does not lead to an extinction of emotional responses or results in a very slow extinction (Paunovic, 1999; Van Rooij et al., 2015; Perusini et al., 2016).

Finally, it is important pointing out that the standard classical conditioning model is unable able to explain these psychopathologies, since it does not account for UCS revaluation nor for the conditions which lead to the prediction error to be equal to zero while an emotional response greater than zero occurs.

**Figure 6:**
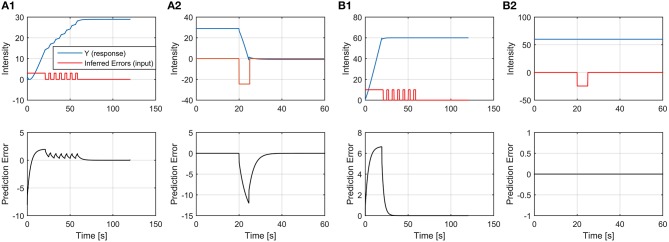
**Simulations of emotional responses determined by prediction errors based on inference learning**. **(A1)** A stressful (non-traumatic) reactive response acquisition: the OFC determines a train of inferred prediction errors which are sent to the amygdala, based on information flux processing. More specifically, the subject consciously expected a negative outcome (i.e., Y expected within the OFC is greater than zero) and the inferred negative outcome is not extreme or traumatic, so that no saturation level of the emotional response, nor hippocampus impairment occur. **(A2)** Physiological devaluation of the previously learned response: the subject is exposed to a stressful-related cue but in a safe context; the inhibition (i.e., deflation) of the emotional reaction determines the devaluation of the response in the safe context. **(B1)** Traumatic response acquisition: inferred prediction errors generate a response which grows at relatively high level, causing both amygdala response saturation and hippocampus impairment. In this case the subject does not expect such an intense negative outcome (i.e., Y expected within the OFC is negligible with respect to the inferred outcome) and/or the inferred outcome is extremely intense. **(B2)** The traumatic response cannot be devaluated since the inhibitory inferred prediction error due to the detection of a safe context is not sufficient to determine a response reduction. This can be determined by two main phenomena: (1) the hippocampus impairment during the traumatic event blocked the encoding of the contextual information, moreover, for this reason, some contextual stimuli have been misattributed as causal sources of stimulation and coded within the amygdala; (2) the weak inhibition due to the safe context is smaller than the degree of saturation reached by the amygdala during the trauma exposure. (*Prediction error* patterns represent the time course of the difference between the expected (within the OFC) and the experienced (within the CNS) responses). Parameter values: model M4, α = 0.4; *K* = 0.5; τ = 2*s*; saturation level for *Y*_*expected*_(*amygdala*) = 150 (which determines a reactive response saturation equals to 60); the level of OFC expectation before the stimulation is equal to 8 in **(A1)** and to 1 in **(B1)**; the inferred outcome is equal to 10 in both **(A1, B1)**.

Figure 6 show results of simulations performed assuming a traumatic and a non-traumatic stimulation. In the former the amygdala response saturation and hippocampus impairment occurred, since the prediction errors due to the difference between the inferred and the expected responses are relatively intense (e.g., death danger). In order to simulate a non-traumatic scenario we have assumed that the expected outcome within the OFC was greater than in the traumatic case, so that the resulting inferred error signals are smaller. Otherwise, it can be assumed that the prediction errors are smaller because the stimulation is less stressful. Nevertheless, assuming a given stressful stimulation, the model suggests that if the expected response within the OFC (i.e., the conscious expectation) is high, the reached response intensity under the stimulation is lower than in the case of a lower expected outcome, and the further extinction is facilitated.

#### 3.2.3. Inextinguishability due to misattribution of a reactive source of emotional stimulation

In order to show how a reactive misattribution leads to a resistant-to-extinction response let us consider the model M1 without H9, and focus in a thought experiment in which multiple trials with interaction between a source of stimulation and a subject occur. Furthermore, let us assume that the elicited response is misattributed to another source of stimulation. In the following we assume, without any loss of generality, that the misattributed source of stimulation is initially *neutral* (i.e., it does not elicit an active or a reactive emotional response). However, if the misattributed source of stimulation is not neutral but elicits a response, the response elicited during the misattribution process will result from the superposition of the actual source response with the previous non-attributed emotional state (Zillmann, 1971). For this reason, in this case the previous non-attributed emotional state “energizes” the actual source.

The emotional misattribution process encompasses the following three mutually exclusive cases:

- Misattribution occurs in the presence of an active stimulation.
- Misattribution occurs in the presence of a residual (i.e., passive) response decay only (in other words, no active or reactive responses are elicited), in this case the misattribution trial follows the elicitation trial and occurs during the excitation decay. This case is known in literature as *transfer paradigm* (described in the Hullian *drive theory* Hull, 1943) or also as *excitation transfer* (Zillmann, 1971; Zillmann et al., 1972; Bunce et al., 1993), and refers to the influence of a prior episode of arousal on subsequent emotional responses.
- Misattribution occurs when a purely reactive source of stimulation is eliciting the subject, so that the associated response is purely reactive.

In this last cited case, of our interest here, the response in the first trial (when the misattribution is occurring), denoted *i*_*U*_, is due to a purely reactive response elicited by an unrevealed or confused source (e.g., a subliminal emotional picture stimulation Esteves et al., 1994; Mayer and Merckelbach, 1999; Glascher and Adolphs, 2003). Hence, the response attributed to the new source (because of the misattribution) is equal to *i*_*U*_, which, in this case, coincides with the response that the amygdala is already eliciting within the CNS. More specifically, since *i*_*U*_ represents the CNS response eliciting by the amygdala, the intensity of the amygdala response (and of the expected response) can be expressed as *i*_*U*_ ∕ α (see Figures 1, 2 and Equation 3). Furthermore, the prediction error in the first trial, which is equal to *i*_*U*_, is sent to the amygdala, and, hence, the overall expected response encoded within the BLA will be due to the sum of the prediction error and the response already elicited by the amygdala, that is *i*_*U*_ ∕ α +*i*_*U*_. Hence, during the second trial, the exposure to the new source determines the reactive response α(*i*_*U*_ ∕ α +*i*_*U*_) which is greater than the expected value previously stored within OFC (that was *i*_*U*_), this leads to the computation of a new prediction error equal to α·*i*_*U*_.

Following this line of reasoning it is easy to prove that the reactive response can be expressed

(39) $$\begin{array}{l} y_{n}=i_{U}+i_{U}\overset{n-1}{\underset{k=1}{\sum }}\alpha^{k} \end{array}$$

after *n* exposure trials, so that the reactive response asymptotically converges to

(40) $$\begin{array}{l} y_{\infty }=\frac{i_{U}}{1-\alpha }. \end{array}$$

Nevertheless, as it is shown is Section 2.2.6 an UCS can be attributed only partially through a statistical inference; this means that in real situations only a portion of the entire response is attributed to the new misattributed UCS, and that successive misattribution trials can improve the attributed response. After some trials in which an emotional picture (UCS1) is paired with a previously neutral picture (CS) the misattribution process, when occurs (since it is stochastic), leads to an overall reactive (and expected) response associated to CS which is comprises between 0 and *i*_*U*_ ∕ (1 − α) (see Equation 40).

A concrete example of such an effect is the EC through *implicit misattribution* (Jones et al., 2009; Hutter and Sweldens, 2013).

Evaluative Conditioning (EC) represents the formation (or change) of the valence of a stimulus, called CS, originating from a prior pairing of the CS itself with another stimulus, called UCS (Baeyens et al., 1993; De Houwer et al., 2001; Jones et al., 2009; Hofmann et al., 2010; Gast et al., 2012; Hutter and Sweldens, 2013); unlike Pavlovian conditioning, a CS response acquired through EC seems to be resistant to extinction (Baeyens et al., 2005).

In Jones et al. (2009) it is shown that, according to the implicit misattribution model, responses to UCSs can be misattributed without awareness to the CS, and that the implicit misattribution depends on *source confusability*. More specifically, the subject may confuse which multiple occurring stimuli in her environment is evoking the evaluative response.

Furthermore, manipulations of the variables related to the potential for the misattribution of an evaluation, (i.e., the source confusability) show that greater EC occurs with an higher degree of confusability (Jones et al., 2009).

This result is also supported by Baeyens et al. (1993), who found that EC was not sensitive to the degree of statistical contingency between the CS and the UCS (as happens in classical conditioning), but EC should increased with the absolute number of pairings because each provides an opportunity for misattribution, and such misattributions could act cumulatively (Jones et al., 2009).

In conclusion, our model based on prediction errors explains, from a quantitative perspective, the EC phenomena and post-predicts the above cited results.

#### 3.2.4. Predictions and results of the discrete model

##### 3.2.4.1. Classical conditioning

M3 model predicts all the relevant results predicted by R-W model for CC (see Miller et al., 1995), since, starting from stochastic hebbian plasticity hypothesis for CS-UCS synaptic connection, the model results in an extended version of the R-W model where, in addiction, accounts for the implicit UCS revaluation. In other words, neglecting UCS revaluation M3 coincides with the R-W model. Moreover, M3 is able to quantitatively justify some experimental results not predictable with R-W model, such as *the dependence of asymptotic responding on CS intensity and US intensity* (Young et al., 1976; see also Miller et al., 1995 and articles therein). Indeed, Equations (30 and 31) show that the CR intensity influences the intensity of the unconditioned response, since, even if the changes of *i*_*R*_ over successive trials could be really small, the asymptotic value of *i*_*R*_ is α*X*∕(1 − α), which is greater than the initial value α*X*. This leads to the conclusion that, since the value of the parameter α is influenced by the selected CS (if the impact of other factors, such as internal physiological states and the selected UCS, is deemed constant), different CSs may result in distinct asymptotic values of *i*_*R*_ (and consequently of *y*_*CS*_; see Equation 31).

Moreover, M3 provides a more general and accurate description of the emotional response during conditioning than the original R-W model for different reasons. First of all, it includes the contributions of both the active response (*X*) due to the elicitation of the UCS and the reactive (self-induced) response associated with the UCS representation within the BLA (*i*_*R*_). Moreover, the recursive equations describing it are *causal* unlike those representing the R-W model. Note that causality ensures that the currently computed response depends only on the past and present values of the stimulus and the response itself, but not on their future values; unluckily, this does not occur for the Rescorla-Wagner model since the evaluation of the current response requires the knowledge of the final asymptotic response, which is actually unknown to the brain. Finally, in our model the CS-UCS synaptic strength and the consequent UCS inflation are jointly considered: the model shows that classical conditioning learning influences the reactive response associated with the paired UCS; this is due to the misattribution of the CR contribution forward the UCS response.

Furthermore, we argue that the model (M1-3) motivates also the *spontaneous recovery* (Miller et al., 1995) and the *conditioned inhibition and all the related phenomena* (such as the *failure of the extinction of conditioned inhibition through non-reinforced presentations of the inhibitor*; DeVito and Fowler, 1987; Harris et al., 2014), which cannot be described in terms of classical conditioning nor TD models (the mathematical demonstrations are not reported in this manuscript).

##### 3.2.4.2. Dopamine neurons activity predictions and relation between existing models

The derived discrete model (M1-3) explain the experimental measures on the activity of dopamine neurons within the VTA (see Schultz, 2002 and articles therein). Indeed, (1) before CC learning a rewarding UCS perception elicits a reactive response; (2) during CC acquisition dopamine neurons respond progressively to the onset of the CS and no more to the UCS; (3) if an UCS stimulation occurs unexpectedly the dopamine neurons activity is physically increased; (4) dopamine neurons encoded the difference between the current reward and an exponentially weighted average of previous rewards (Bayer and Glimcher, 2005). Our model is able to justify such dopamine neurons behaviors, more specifically, whenever the CS-UCS connection strength increase the reactive response (*i*_*R*_) associated with the given UCS can be activated by the CS perception through the synaptic connection ω_*CS*−*UCS*_ (see Figure 2; nevertheless, before any cue association, only the UCS perception is able to activate the reactive response (all the intermediate situations in which 0 < ω_*CS*−*UCS*_ < 1 are also predicted). Moreover, if successive reward stimulations occur the error signal at each trial is effectively computed as an exponentially weighted average of previous outcomes; furthermore whenever an unexpected reward occurs an adding reactive response due to contrast effect is triggered. Such a contrast reactive response determines an error signal which update the UCS expected value only the first time it occurs (see Section 2.5.1), and this assures that an unbounded increasing of the UCS associated value does not occur. The last reasoning supports the idea that the reactive contrast effect is different than prediction error and it represents a type of reactive response. Indeed, other than reward prediction errors, different types of dopamine neural coding exists (Bromberg-Martin et al., 2010). More specifically, dopamine neurons can in some cases compute prediction errors, and in other cases code or compute reactive responses associated with given stimuli (CSs or UCSs) in order to promote attention and specific (approaching or avoiding) behaviors. The same function is performed by contrast effects which have to focus attention toward the stimulator promoting further learning (for instance, discovering a specific cue which may in the future predict such an unexpected stimulation). One of the difference between our theory and TD models for reinforcement learning (Schultz et al., 1997; Schultz, 1998) is that the latter assume that dopamine neurons encode only prediction errors (and does not take into consideration nor define the term “reactive response”), and that learning can occur only in the presence of a prediction error. Nonetheless, there are evidences which disagree with TD models: for instance, the occurrence of associative learning between two neutral stimuli (the so called, *sensory pre-conditioning;* Young et al., 1998; Sadacca et al., 2016) or the evidence obtained by fMRI studies in which the conditioned acquisition, or, in other words, an increase of the CS-UCS contingency occurs even in presence of a negative prediction error due to the concurrent deflation (decrease) of the UCR. In practice, if during acquisition (CS-UCS pairings) the intensity of the UCS stimulation is reduced, the CS-UCS connection is still increased but a negative prediction error is computed and updates the expected outcome associated to the UCS (Gottfried and Dolan, 2004). Also the opposite situation may occur: during conditioning extinction a positive prediction error due to UCS inflation can be obtained. Such discrepancies originate from the fact that TD (and R-W) models do not account for the two main different types of learning (CC and, more generally, the *statistical and inferential learning* and *UCS revaluation driven by prediction errors*, see Figure 1). In particular, the inferential and statistical learning creates a so called *model of the world* (Doll et al., 2012), and it is not driven by errors but it occurs through statistical (e.g., Bayesian) inference. Such an inference about the statistical contingencies and causalities can be performed by the hippocampus (and even directly by the amygdala) for low level information processing, or by OFC whenever complex pattern or higher level information have to be analyzed (see Figure 1). Furthermore, as we have previously shown (see Equation 24), the statistical inference contribution and the prediction error based contribution (also called *model-free* contribution; Doll et al., 2012) are taken into consideration by the brain for the computation of the expected response depending on the degree of certainty (belief) of each of the two components.

In conclusion, our model post-predicts the most relevant phenomena related to learning and dopamine neurons, moreover it predicts further important related phenomena with respect to existing learning models.

### 3.3. Validation, interpretation, and applicability of the model

#### 3.3.1. Validation of the discrete model

The discrete model parameters (i.e., α, *K*, and the filtering coefficient, together with the estimation of the induced *x*) can be estimated, for every emotional component, inducing specific stimulation trials while neuronal activity is monitored (e.g., by fMRI or direct neurons activity recording). For instance increasing intensity electric shock delivery can be performed estimating the parameters valid for fear and anxiety related emotional responses, and from unexpected stimulations the contrast parameter*K* can be estimated; furthermore, rewarding stimulations can be induced in order to estimate the parameters valid for the dopamine neuron populations (e.g., in VTA).

#### 3.3.2. Discrete model applications

The discrete model can be adopted in different psychological paradigms and experiments other than the study of dopamine neurons behavior. For instance, it is well known from the literature (Bechara et al., 1994) that patients with damaged OFC (and PFC) perform poorly at the Iowa Gambling Task (IGT). It is thought that such patients cannot learn from previous emotional error signals, and, in line with this reasoning the structure derived in Figure 1 shows that if OFC is damaged the emotional prediction error computation is compromised. Our model can be applied to the study of IGT, more precisely, a given deck of cards represents an UCS whose average stimulation has to be estimated, and the single cards represent specific emotional stimulations (in this case purely reactive, since no active/energy based stimulations occur). Firstly the model parameters have to be inferred by successive stimulations and neuronal activity measurements (e.g., by fMRI). Such parameters are: α, *K*, *h*_*k*_(which represents a unique exponential coefficient considering an exponential weight average filtering), *x*_*i*_(*i* = 1, …*R*) where R represents the number of the possible card outcomes and *x*_*i*_ represents the emotional response associated to the i-th stimulation card (e.g., a gain of 50$). Such parameters have to be estimated monitoring the neuronal activity while, from a unique deck, successive cards (with random order) are discovered by the subject (for instance, the subject perceive the sequence +50$, +100$, -50$…). Once known the parameters related to a given subject (or to a group of subjects), the model can predict the performance of that subject at IGT, provided that the sequence pattern (i.e., cards sequences) are given. More specifically, some patients can perform poorly because an altered *K* parameter, others because an altered α parameter, or because an unbalanced reactive response associated with positive (rewarding) with respect to the negative cards (*x*_*i*_), and so on. For instance similar studies have been proposed for patients with Parkinson's disease (Zaghloul et al., 2009).

#### 3.3.3. Validation of the continuous time model

M4 can be validated in different scenarios. For instance the time constant τ_2_ related to the filtering process and the parameterα (for a target population) can be estimated applying a (constant) direct neuronal stimulation to the target population and measuring the overall response over time (this could be accomplished adopting optogenetic manipulation technology (Redondo et al., 2014), performing the so called *step response measure*). In this scenario the asymptotic value is related to α and to the direct stimulation by the relation seen in Equation (13), while the rise time is related to τ_2_. Furthermore, the model can be tested applying a time varying stimulation over time while recording the overall response. Finally, even a time varying function representing the error signal dynamics can be directly applied to the neuronal population involved in error signal computations (e.g., the VTA) while the overall response dynamics is recorded in the target population (e.g., in the NAcc), similarly as performed in Chang et al. (2016).

#### 3.3.4. Applications and modulation (increasing/decreasing) of emotional responses

The derived model can be adopted for the study of the emotional dynamics during a continuous stimulation. A practical example is music, in which complex hierarchical patterns of acoustic sound successions (which involve inferential learning/pattern recognition) together with continuous modulation of contrast effects are exploited to evoke specific emotional responses. It is well known that music is able to evoke emotions, for instance, violating expectations or shifting in time the rewards in a balanced mechanism based on frustration (i.e., tension, as a state of dissonance, instability and uncertainty Huston et al., 2015) and satisfaction (resolution toward consonant and stable sounds experienced as pleasurable; Koelsch, 2014). Violation or retardation in resolution produces a tension increase which may result in a successive stronger satisfaction during resolution (Huston et al., 2015). However, it has to remember that the specific mapping function between the features of a given physical source, which drives the energy flux, and the corresponding active emotional response induced by it (i.e., *x*(*t*) or *x*_*n*_, which represents the mean firing rates of a given neuronal population elicited by the energy flux) has to be determined. If this function is known, the physical features of the source can be controlled in a way to generate specific dynamics in the active response; this, in turn, results in the generation of designed emotional reactive responses.

##### 3.3.4.1. Artificial emotional modulation and production of resistant-to-extinction responses

Our theoretical findings suggest that the “inertial nature” of the *emotional dynamic tracking system* can be exploited to originate a resistant-to-extinction emotional reaction; this, in turn, may be exploited to increase (decrease) an emotional response until a saturation level (zero). In the following is explained how this can be obtained.

Starting from Equation (38) it is possible performing numerical optimization procedures to find *x*(*t*) patterns which determine a CNS response (denoted *y*(*t*)) such that an *y*(*t*) different from zero occurs while a “very close to zero” prediction error (*e*(*t*)) occurs together with the condition that *x*(*t*) is close to zero, within a sufficient long time interval. The functional to be optimized has to involve all the above mentioned conditions. Despite the fact the so obtained reactive response could be relatively small, the stimulus which is associated to such a pattern of stimulation will acquire a resistant-to-extinction response; furthermore, since an emotional reaction (even if small) is triggered, even in absence of an active stimulation (since it is resistant-to-extinction) the presentation of a new input stimulation function (i.e., *x*(*t*)), with the same dynamics of the previous one, will permit to obtain an increasing of the response, exploiting a summation effect (see the *integration property* and Equation 2). Hence, with a limited (and periodical) dynamics of the input *x*(*t*) is possible to obtain an “unlimitedly” increasing emotional reaction exploiting the inertial nature of the emotional system. In order to test the hypothesis, after having obtained the desired *x*(*t*) function from numerical optimizations, such a function can be applied to CNS emotional population (e.g., the anterior cingulate regions, or rewarding brain regions such as NAcc) through optogenetic manipulations (Redondo et al., 2014), or through direct electric neuronal stimulation, while recordings the increase of the overall population emotional response over time. We argue that at every application of the optimized *x*(*t*) there is a probability greater than zero that the “inextinguishability effect” takes place, adding a contribution to the previously accumulated resistant-to-extinction response.

#### 3.3.5. Inducing traumatic (saturated) responses: testing the model

On the basis of the analysis and models developed in the previous sections, we argue it is possible to induce traumatic emotional responses in a laboratory through optogenetic manipulations. More specifically, the misattribution effect and the implicit UCS revaluation can be exploited in an iterative framework until a saturation level will be reached. It is proposed the following procedure: (1) the animal has to infer that a given electric shock device (UCS1) is the causal source of pain; in particular, at every stimulation trial UCS1 has to be perceived by the animal, emitting also a brief specific acoustic tone (CS1, which will serve only as “probe” to test the response inextinguishability at the end of the inductive process) just before the occurring of the electric shock stimulation. It is important to note that every time that UCS1 is presented to the animal, it will elicit the electric shock. (2) Some stimulation trials have to occur in order to reach the asymptotic response *y* = *x*∕(1 − α), provided that *x* represents the active component. (3) A second pain stimulator device (UCS2), different from UCS1 (for instance a device producing pain by heat shock can be adopted) has to be presented to the animal exactly as UCS1. (4) Neuronal representations (memory engrams) of UCS1 and UCS2 have to be detected and labeled within the BLA. At this point it is important to note that two distinct reactive responses, *i_R_1__*, *i_R_2__*(such that *i_R_1__* = α*x*_1_ ∕ (1 − α); *i*_*R*_2__ = α*x*_2_ ∕ (1 − α)), have been associated with the BLA memory engrams of UCS1 and UCS2 respectively, so that it is possible to activate such reactive responses by optical stimulation of the associated engram cells (Ramirez et al., 2015). (5) In successive UCS1 stimulation trials, the BLA memory engram associated with UCS2 has to be optically stimulated in order to induce an UCS1 revaluation determined by the sum of the active (electric shock elivery, denoted *x*), the UCS1 reactive response *i*_*R*_1__ and the reactive response associated with UCS2, *i*_*R*_2__. The above mentioned revaluation occurs since the overall response (active and reactive) will be fully attributed to UCS1 (in other words, a misattribution does occur). (6) Some stimulation trials have to be performed in order to reach the new asymptotic response attributed to UCS1, which will be equal to:

(41) $$\begin{array}{l} y_{UCS1}^{1-\infty }=x_{1}+i_{R_{1}}+\frac{i_{R_{2}}}{1-\alpha }=x_{1}+i_{R_{1}}+\frac{\alpha i_{R_{2}}}{1-\alpha }+i_{R_{2}} \end{array}$$

where, $y_{UCS1}^{1-\infty }$ represents the asymptotic UCR1 at the end of the first iterative procedure; (7) The UCS2 stimulates the animal, while optical stimulation of the UCS1 memory engram occurs, so that the UCR2 in the first trial of the second iterative procedure can be expressed as:

(42) $$\begin{array}{l} y_{UCS2}^{2-1}=x_{2}+i_{R_{2}}+i_{R_{1}}. \end{array}$$

It is worth noting that, at this stage, the reactive response *i*_*R*_1__ has been increased during the first iterative procedure, and its value was increased from α*x*_1_ ∕ (1 − α) to (see Equation 41):

(43) $$\begin{array}{l} i_{R_{1}}^{1-\infty }=\frac{\alpha x_{1}}{1-\alpha }+\frac{\alpha i_{R_{2}}}{1-\alpha }=\frac{\alpha x_{1}}{1-\alpha }+\frac{\alpha^{2}x}{{\left(1-\alpha \right)}^{2}}. \end{array}$$

If, without any loss of generality, it is assumed that *x*_1_ = *x*_2_ = *x* and that α = 0.5 in order to simplify the computations, the UCR2 asymptotic value at the end of the second iterative procedure can be expressed as:

(44) $$\begin{array}{l} y_{UCS2}^{2-\infty }=x+i_{R_{2}}+\frac{i_{R_{1}}^{1-\infty }}{1-\alpha }=x+x+4x=6x \end{array}$$

(8) procedures 6 and 7 are repeated iteratively, increasing *i*_*R*_1__ and *i*_*R*_2__at every stimulation trial; we named this procedure *iterative climbing*, since the derived protocol resembles a climbing performed by leaning iteratively between UCS1 and UCS2. It is easy to verify by induction that the process leads to a response which diverge to infinity (i.e., $y_{UCS1}^{\infty -\infty }\to \infty$). In practice, it is expected that when a saturation threshold is reached, the error signal will be zero and no more response increases can occur. In such a situation the emotional response will be resistant-to-extinction and the simple presentation of the UCS1 with the probe CS1 (without active stimulation) to the animal will produce the traumatic reaction. Indeed, UCS1 (and CS1) represent the traumatic triggering cues; it is also expected that the only CS1 presentation is able to trigger such a traumatic response like in PTSD patients.

## 4. Discussion

In this manuscript a system computational model of emotional learning has been developed. The model shows the differentiation (and the relations) between statistical inference learning (e.g., CC) and implicit UCS revaluation, and provides various new insights on well known psychophysiological phenomena and psychiatric diseases, and new ideas for further research. One of its most interesting implications is represented by the identification of well defined mathematical and neurophysiological conditions ensuring the inextinguishability of specific emotional reactions. In particular, it allows us to establish the following four different mechanisms through which a stimulus can produce a resistant-to-extinction emotional reaction: (1) misattribution of a reactive response; (2) classical conditioning of a stimulus to a purely reactive UCS; (3) saturation of emotional response together with hippocampus impairment (also reproducible through optogenetic manipulations exploiting the *iterative climbing procedure*); (4) the exploitation of the inertial dynamics of emotional system on a continuous time scale. Further relevant contributions are represented by the proof that the Rescorla-Wagner model for classical conditioning can be obtained as a special case of the proposed model; the derivation of a new model for conditioning, which accounts for the implicit UCS revaluation and that is able to quantitatively describe important experimental results, which are unpredictable by existing classical conditioning models (including the TD model).

Our result paves the way for various new research activities. First of all, various potential applications of our theory can be envisaged in the hot research area concerning the study (and the manipulation) of animal behaviors, emotional reactions and decision making, since our model permits to infer specific parameters involved in emotional induced responses under decision, which are known to influence (or even drive) human decisions (see Bechara et al., 1994).

A further relevant research topic concerns the applications of our model of emotional learning on a continuous time scale. Generally speaking, this model could be exploited to analyze the emotional reaction generated by any stimulation which varies continuously over time (e.g., a time-varying acoustic source of stimulation, such as music, or even a purely reactive emotional induction, such as a succession of emotional pictures, or a movie).

Finally, it is important to mention that our theoretical framework can be exploited for the development of animal psychophysiological experimental models; these, in turn, can potentially provide new insights into emotion-related phenomena and pathologies.

## Author contributions

LP and SR wrote the main manuscript text; SR prepared Figures 1–6. All authors reviewed the manuscript.

### Conflict of interest statement

The authors declare that the research was conducted in the absence of any commercial or financial relationships that could be construed as a potential conflict of interest.
