# Supplementary material for: A System Computational Model of Implicit Emotional Learning
Source: Front Comput Neurosci. 2016 Jun 14;10:54. doi: 10.3389/fncom.2016.00054 (PMC4906031; doi:10.3389/fncom.2016.00054)
Supplement: Supplementary file 1 [file Presentation1.PDF]

Supplementary Material: A System Computational Model of Implicit Emotional Learning

Luca Puviani*, Sidita Rama
*Correspondence:
Luca Puviani:
luca.puviani@unimore.it

1 SUPPLEMENTARY DATA

1.1 Emotional system stability theorem

Theorem: *Necessary condition for the stability of the emotional system is that the emotional response associated to a given UCS is a fraction of the expected (predicted) response.*

PROOF. ***Proof by contradiction (reductio ad absurdum)***

Hypothesis 1: The emotional response associated with a given UCS coincides with the expected (predicted) outcome, and the predicted outcome converges to the experienced outcome.

Hypotheses 2: The properties integration, reactive mimicking and emotional system stability hold.

Hypotheses 3: Cognitive information processing are avoided (in other words only automatic and implicit evaluation processes are considered).

Hypotheses 4: A single emotional component is considered. This does not imply any loss of the generality since it is possible to reconstruct the entire emotional response computing the summation of the involved neuronal populations (see Eq. (1) of the main manuscript) provided that only the components belonging to the emotional system are considered).

Hypothesis 1 asserts that the emotional response induced reactively by the UCS coincides with the expected outcome; furthermore, the predicting signal converges (through a learning process) to the actual experienced elicitation. The last assumption has been formulated to include a more general scenario than that considering the expected outcome coincides with the last experienced outcome. From a mathematical viewpoint, the expected outcome can be computed using any supervised learning method (or, alternatively, TD methods in Sutton, 1988) in which the predicted outcome is evaluated on the basis of the past m predictions (i.e., of the predicted outcomes in the last m trials) and of the actual outcome, minimizing the error between the prediction and the experienced outcome. Otherwise it can be assumed that the predicted outcome coincides with the last experienced outcome.

1. Let the UCS be an active source of stimulation (e.g. a painful stimulator or a drug administration whose target belongs to the emotional system).
2. UCS perception and the successive active stimulation takes place on successive trials, so that it exerts first a reactive response an successively an active elicitation (i.e., it elicits an active response, denoted

x). During the first trial the response is exclusively due to the active contribution, that is $y_1 = x$, since no reactive response can be elicited because the UCS was unknown to the emotional system before the first active elicitation. After the first trial (for instance, during the UCS perception in the second trial), the predicted (which coincides with the reactive) response, called $y_{predicted,1}$, is computed.

3. In the second trial, after the UCS perception, the predicted outcome ($y_{predicted,1}$) adds up to the successive UCS active elicitation, so that the outcome can be expressed as $y_2 = y_{expected,1} + x$. Furthermore, since $y_{predicted,1}$ does not coincide with the actual experienced outcome, the new prediction $y_{predicted,2}$ is computed after the second trial; it can be easily proved that $y_{predicted,2} > y_{predicted,1}$ (since the experienced outcome has been strengthened and the error signal has to be minimized).
4. In the third trial the experienced outcome can be written as $y_3 = y_{predicted,2} + x$; since $y_3 > y_2 \geq y_{predicted,2}$ a new value for the predicted response is computed, called $y_{predicted,3}$, such that $y_{predicted,3} > y_{predicted,2}$.
5. In the n-th trial the outcome can be expressed as $y_n = y_{predicted,n-1} + x$; it is easy to prove that $y_n > y_{n-1} \geq y_{predicted,n-1}$. Moreover, if the number of trials tends to infinity, the response grows indefinitely (i.e., $\lim_{n\to\infty} y_n = \infty$).
6. The last statement is absurd, as it contradicts Hypothesis 2, in particular it contradicts the stability system principle.

1.2 Classical conditioning and UCS revaluation in the discrete-time scale

In this Section a discrete model of classical conditioning is derived through a thought experiment, where a sequence of trials, involving CS-UCS-subject interactions, is analysed. Our derivation relies on the assumption that the CS-UCS synaptic connections are governed by the mechanisms of *stochastic Hebbian plasticity* (Amit and Fusi, 1994; Fusi, 2002; Fusi and Abbott, 2007; Hebb, 1949; Soltani and Wang, 2006, 2010). In these trials we assume that: a) the source of stimulation (UCS) has been correctly coded by the emotional system of the subject, and that the expected response associated with the UCS representation is equal to X, so that the associated reactive response i_R can be assumed to be equal to $\alpha \cdot X$; b) a new cue (denoted CS) becomes paired with the UCS during active stimulations; c) in the first trial the synaptic strength $\omega^{(1)}_{CS-UCS}$ (ranging from 0 to 1, as evidenced below) of the CS-UCS connection (see Fig. 1) is equal to zero (in other words, no connection has been established before the start of the acquisition process); d) the CS remains the same during all the considered trial; e) the UCS elicitation is signalled by the CS presentation only, and not by a prior UCS presentation (for instance, the electric shock device (UCS) is permanently attached to the subject and the shock delivery is signalled exclusively by the CS presentation). In the following analysis the response elicited by the CS, previously named CR, is denoted y_{CS}.

In the first trial, an unexpected active UCS elicitation (X) generates the emotional response:

$$y^{(1)}_{UCS} = X, \tag{1}$$

which is what was expected for the source UCS. However, since the UCS occurs unexpectedly in time, a reactive contribution due to the *contrast effect* (see Section 2.9 in the main text) should be produced. Nevertheless, in Section 2.9 it has been shown that successive unexpected UCS elicitations do not entail the computation of a prediction error, but certainly involve the elicitation of a reactive response due to the contrast effect, (quantified by the product $K \cdot X$). However, without any loss of the generality, this reactive contribution can be neglected; furthermore, it can be noted that, during conditioning trials, this contrast

contribution will vanish (since no “unexpected stimulation” occurs as the CS becomes progressively able to predict the UCS occurrence) and in place of this contribution the reactive response associated with the UCS (i_R) will be elicited as the CS is perceived (see Fig. 1). Even if no error signal has to be estimated for the given UCS during the first trial, the concurrent presence of the CS during the UCS elicitation is sufficient to generate some synaptic connections between the representation of the CS and that of the UCS through the stochastic Hebbian rule. Hence, the strength of the CS-UCS link is potentiated through LTP. In the following it is also assumed that individual plastic synapses exhibit a binary behavior, since they can be in a *depressed state* or in a *potentiated state*. For this reason, the strength of a set of plastic synapses is quantified by the fraction of synapse population in the potentiated state (Amit and Fusi, 1994; Fusi, 2002; Fusi and Abbott, 2007; Soltani and Wang, 2006, 2010); this fraction is called *synaptic strength* and in the n-th trial is denoted as $\omega^{(n)}_{CS-UCS}$ for the set of synapses from the neurons representing the CS stimulus onto the encoding neurons for the UCS. The mechanism through which plastic synapses learn cue-outcome contingencies through stochastic reward-dependent Hebbian modifications is illustrated in Soltani and Wang (2010). In practice, whenever the neurons encoding a given CS are simultaneously elicited at the activation of the UCS neurons, the plastic synapses from CS onto UCS in the depressed state make a transition to the potentiated state with probability $\hat{\alpha}_+$ (this quantity is called *potentiation rate*); otherwise, if the CS perceived without the contingent UCS perception, they make a transition in the reverse direction with probability $\hat{\alpha}_-$ (this quantity is called *depression rate*). It is worth noting that the parameters $\hat{\alpha}_+$ and $\hat{\alpha}_-$ are scalar quantities (each of them would be replaced by a vector having identical components if multiple emotional components were considered in the evaluation of the reactive response) and they are not related to the emotional learning rate α defined in the Sections 2.7 of the main text. On the basis of the plastic probabilistic Hebbian rule illustrated above, in the n-th trial the synaptic strength is updated as

$$\omega^{(n)}_{CS-UCS} = \omega^{(n-1)}_{CS-UCS} + \hat{\alpha}_+ \cdot \left(1 - \omega^{(n-1)}_{CS-UCS}\right) \tag{2}$$

during a conditioned acquisition (through LTP), and as

$$\omega^{(n)}_{CS-UCS} = \omega^{(n-1)}_{CS-UCS} - \hat{\alpha}_- \cdot \omega^{(n-1)}_{CS-UCS} \tag{3}$$

during the extinction phase (through LTD). Note that the second term in the right-hand side of (2) describes the change related to the transition of synapses in the depressed state, since a fraction $\left(1 - \omega^{(n-1)}_{CS-UCS}\right)$ of synapses are potentiated with probability $\hat{\alpha}_+$.

The synaptic strength $\omega^{(n)}_{CS-UCS}$ evaluated on the basis of Eq. (2) can be exploited to assess the response $y^{(n)}_{CS}$ to the presentation of the CS alone in the n-trial; in fact, the intensity of the CR is determined by the product of $\omega^{(n)}_{CS-UCS}$ with the reactive response i_R associated with the paired UCS, i.e. by

$$y^{(n)}_{CS} = \omega^{(n)}_{CS-UCS} \cdot i_R. \tag{4}$$

Then, substituting (2) in (4) yields the expression

$$y^{(n)}_{CS} = i_R \cdot \omega^{(n-1)}_{CS-UCS} + \hat{\alpha}_+ \cdot \left(i_R - i_R \cdot \omega^{(n-1)}_{CS-UCS}\right), \tag{5}$$

which can be easily put in the form

$$y_{CS}^{(n)} = y_{CS}^{(n-1)} + \hat{\alpha}_+ \cdot \left(i_R - y_{CS}^{(n-1)}\right). \tag{6}$$

It is easy to prove that the last formula coincides with the well known Rescorla-Wagner equation for Pavlovian conditioning (Miller et al., Sec 1, p. 365, eq. (1-2)),

$$V_x^{n+1} = V_x^n + \alpha_x \beta_1 \left(\lambda_1 - V_{total}^n\right) \tag{7}$$

for the case in which a single CS is considered; in fact, Eq. (7) is obtained from (6) if $\hat{\alpha}_+$ and i_R are replaced with $\alpha_x \cdot \beta_1$ and λ, respectively, and $y_{CS}^{(n-1)}$ is assumed to represent the *associative strength* V_x^n. Note that the V_{total}^n coincides with the term V_x^n if a single CS (denoted x) is considered; otherwise it represents the sum of the associative strengths of all CSs (including x). If is assumed that CS is composed by N distinct CSs (i.e., a *CSs compound* is considered), the synaptic strength between the compound and its paired UCS can be still computed on the basis of (6). However, in this case, a fraction of the overall strength should be associated with each component of the compound CS (such a fraction depends on the nature of the considered CS and its neural representation). Then, the contribution to the synaptic strength originating from the k-th component (with $k = 1, 2, ..., K$) can be evaluated as (see ((2))

$$\omega_{CS_k-UCS}^{(n)} = \omega_{CS_k-UCS}^{(n-1)} + \hat{\alpha}_k \cdot \left(1 - \omega_{totCS-UCS}^{(n-1)}\right), \tag{8}$$

where $\omega_{CS_k-UCS}^{(n)}$ and $\omega_{totCS-UCS}^{(n-1)}$ represent the synaptic strength originating from the k-th CS and the overall synaptic strength originating from the compound, respectively. The last formula is motivated by the fact that each component shares the same full synaptic connection in reaching the neural representation of the stored UCS. It is also worth mentioning that multiplying both sides of (8) by i_R produces

$$y_{CS}^{(n)} = y_{CS}^{(n-1)} + \hat{\alpha}_+ \cdot \left(i_R - y_{total}^{(n-1)}\right), \tag{9}$$

which represents the Rescorla-Wagner equation for the case of a CS compound (Miller et al., 1995, Sec 1, p. 365, eq. (1-2)). From Eq. (8) it can also inferred that, if a CS or a compound have been conditioned to a UCS, so that their synaptic strength is equal to unity, when a new CS is added to the compound and paired with the given UCS no connection updating can occur, this phenomenon is known as *blocking effect* (Miller et al., 1995).

Nevertheless, the derived model is only an approximation, since it assume that the expected (and hence also the reactive) response associated with the given UCS is constant over successive trials. Nonetheless this is not true, on the basis of the UCS revaluation model derived in the previous sections. Indeed, since the CS-UCS connection strength increases during conditioning acquisition, the reactive response i_R associated with the UCS should also increase. As a matter of fact, the emotional response $y_{UCS}^{(n)}$ in the n-th acquisition trial is due to the elicitation of the CS response (i.e., to $y_{CS}^{(n)} = i_R \cdot \omega_{CS-UCS}^{(n)}$ due to the partial activation of the UCS representation from the CS) and to the active UCS stimulation (X), since the overall response is *attributed* to the UCS (which represents, unlike the CS, a direct source of stimulation). This last claim is supported by the experimental results obtained through optogenetic manipulations (Redondo et al., 2014), which show that a memory engram coding a CS is "emotionally neutral" and could freely associate with different emotional responses through the corresponding UCS representations coded within the BLA. Therefore, it is easy to prove that the associated reactive component i_R grows from the initial value $\alpha \cdot X$ to the value $\alpha \cdot (X + i_R^{(n-1)} \cdot \omega_{CS-UCS}^{(n)})$ because of the prediction error computation. For these reasons,

the process of implicit UCS inflation originates from an indirect contribution of the CS; in fact, the CS, signalling the UCS, is able to elicit the reactive response (i_R; see Fig. 1) associated with the UCS itself. Therefore, i_R does not remain constant over consecutive acquisition trials, as assumed by the original Rescorla-Wagner model, but evolves according to the recursive equation

$$i_R^{(n)} = \alpha \cdot \left(X + i_R^{(n-1)} \cdot \omega_{CS-UCS}^{(n)} \right) \tag{10}$$

The last formula shows that the CR intensity influences the intensity of the unconditioned response. This result is in agreement with some experimental results (Young et al., 1976) (see also Miller et al., 1995 and articles therein), evidencing *the dependence of asymptotic responding on CS intensity and US intensity*. It is also important to point out, however, that the changes of i_R over successive trials could be really small, even if the asymptotic value of i_R is $\alpha X/(1-\alpha)$, which is greater than the initial value αX. This leads to the conclusion that, since the value of the parameter α is influenced by the selected CS (if the impact of other factors, such as internal physiological states and the selected UCS, is deemed constant), different CSs may result in distinct asymptotic values of i_R (and consequently of y_{CS}; see Eq. (4)).

In summary, we propose to adopt a new classical conditioning discrete model, which encompasses the Rescorla-Wagner model and coincides with it only if UCS revaluation is neglected. If a single CS is assumed to simplify the notation, this extended model is described by Eq. (2) when the given CS is paired with an UCS, Eq. (3) when the CS is presented alone, by Eq. (10), which has to be updated only in the CS-UCS pairing trials (since i_R does not vary if the CS is presented alone), and, finally, by the formula

$$y_{CS}^{(n)} = \omega_{CS-UCS}^{(n)} \cdot i_R^{(n-1)}, \tag{11}$$

which expresses the y_{CS} updating.

REFERENCES

Amit, D. and Fusi, S. (1994). Dynamic learning in neural networks with material synapses. *Neural Comput.* , 6, 957–982

Fusi, S. (2002). Hebbian spike-driven synaptic plasticity for learning patterns of mean firing rates. *Biol. Cybern.* 87, 459–470

Fusi, S. and Abbott, L. (2007). Limits on the memory storage capacity of bounded synapses. *Nat. Neurosci.* 10, 485–493

Hebb, D. (1949). *The organization of behavior* (New York: Wiley)

Miller, R., Barnet, R., and Grahame, N. (1995). Assessment of the rescorla-wagner model. *Psychol. Bull.* 117, 363–386

Redondo, R., Kim, J., Arons, A., Ramirez, S., Liu, X., and Tonegawa, S. (2014). Bidirectional switch of the valence associated with a hippocampal contextual memory engram. *Nature* 513, 426–430

Soltani, A. and Wang, X. (2006). A biophysically based neural model of matching law behavior: melioration by stochastic synapses. *J. Neurosci.* 26, 3731–3744

Soltani, A. and Wang, X. (2010). Synaptic computation underlying probabilistic inference. *Nat. Neurosci.* 13, 112–119

Sutton, R. (1988). Learning to predict by the methods of temporal differences. *Machine Learning* , 3, 9–44.

Young, R., Cegavske, C., and Thompson, R. (1976). Tone-induced changes in excitability of abducens motoneurons and of the reflex path of nictitating membrane response in rabbit (oryctolagus cuniculus). *J. Comp. Physiol. Psychol.* 90, 424–434
